# Enhancers compete with a long non-coding RNA for regulation of the *Kcnq1* domain

**DOI:** 10.1093/nar/gku1324

**Published:** 2014-12-24

**Authors:** Bryant M. Schultz, Gwendolyn A. Gallicio, Matteo Cesaroni, Lena N. Lupey, Nora Engel

**Affiliations:** Fels Institute for Cancer Research and Molecular Biology, Temple University School of Medicine, Philadelphia, PA 19140, USA

## Abstract

The imprinted *Kcnq1* domain contains a differentially methylated region (*KvDMR*) in intron 11 of *Kcnq1*. The *Kcnq1ot1* non-coding RNA emerges from the unmethylated paternal *KvDMR* in antisense direction, resulting in *cis*-repression of neighboring genes. The *KvDMR* encompasses the *Kcnq1ot1* promoter, *CTCF* sites and other DNA elements, whose individual contribution to regulation of the endogenous domain is unknown. We find that paternal inheritance of a deletion of the minimal *Kcnq1ot1* promoter derepresses the upstream *Cdkn1c* gene. Surprisingly, *Kcnq1ot1* transcripts continue to emerge from alternative sites, evidence that silencing depends, not on the ncRNA, but on the promoter sequence. Detailed analyses of *Kcnq1ot* during cardiogenesis show substantial chromatin reorganization coinciding with discontinuous RNA production in both wild-type and mutant mice, with loss of imprinting. We show that *CTCF* binds to both methylated and unmethylated alleles of the KvDMR. Furthermore, we report a multitude of enhancers within the *Kcnq1ot1* region, and present conformational dynamics of a novel heart enhancer engaged in *Kcnq1* expression. Our results have important implications on tissue-specific imprinting patterns and how transcriptional mechanisms compete to maximize the expression of vital genes, in addition to shifting our perception on the role of the long ncRNA in regulating this imprinted domain.

## INTRODUCTION

**Figure 1.**
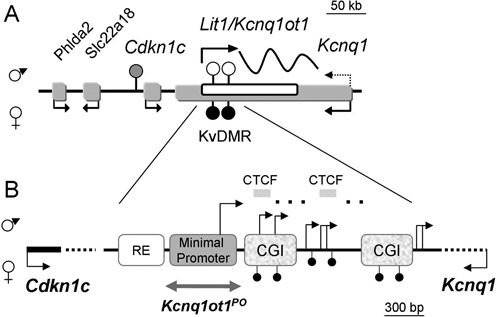
*Kcnq1* domain: expression and imprinting features. (**A**) Overview of the genes within the *Kcnq1* domain exhibiting monoallelic expression in the embryo. Paternal and maternal expressions are indicated with arrows above and below the sequences, respectively. Arrows show the direction of transcription. Dotted arrow emerging from *Kcnq1* indicates transition to biallelic expression in mid-gestation. Filled and unfilled circles indicate methylated and unmethylated regions, respectively; hatched circle represents a methylation mark acquired after fertilization. Scale is indicated above the domain. (**B**) Schematic of the ∼4300 bp KvDMR and its regulatory features. RE, regulatory element; CGI, CpG islands; hatched bars, CTCF sites; broken arrows indicate the transcriptional start sites reported previously (16,19,24), some of which are tissue-specific. Gray double-headed arrow below the minimal promoter represents the region deleted in this report. Scale is indicated below the schematic.

Thousands of long non-coding (lnc) RNAs are produced by the mammalian genome, but few are larger than 10–20 kb. *Kcnq1ot1* has been described historically as a lncRNA of ∼90 kb, emerging from intron 11 of *Kcnq1* in the antisense direction (1,2). In the early embryo, *Kcnq1ot1* is only expressed paternally and silences three upstream genes, *Cdkn1c, Slc22a18* and *Phlda2*, acting in *cis* (reviewed in (3,4)) (Figure 1A). The mechanism by which the ncRNA regulates its neighboring genes has not been clearly established, although two hypotheses have been put forward: one proposing a direct action of the *Kcnq1ot* molecule by spreading and recruiting repressive factors (5); and the other, suggesting that regulatory DNA elements exposed by the act of transcription of the ncRNA are responsible for the silencing (6,7).

Although it was initially assumed that *Kcnq1ot1* also silenced its sense counterpart, the *Kcnq1* gene, two observations led us to reassess how the ncRNA regulates *Kcnq1*: (i) studies in whole embryos and neonatal mouse tissues revealed that expression of *Kcnq1* transitions to a biallelic mode after mid-gestation (8); (ii) detailed expression and conformational profiles of the developing heart showed that the loss of *Kcnq1* imprinting coincides with activation of strong cardiac-specific enhancers that physically engage with the *Kcnq1* promoter (9). Based on these observations, we suggested that enhancer-driven expression could successfully compete and override the silencing effects of *Kcnq1ot1* transcription on the paternal *Kcnq1* allele.

Defects in KCNQ1 are responsible for congenital long-QT syndrome, with cardiac phenotypes of different severities. *Cdkn1c*, the closest gene silenced on the paternal chromosome, is also important for regulating proliferation during heart development (10,11), specifically at E9.5–13.5, probably acting as a downstream target of *Bmp10* signaling.

The main *Kcnq1ot1* promoter is part of a region designated as the *KvDMR* (Figure 1B). Previous gene targeting studies have deleted the entire *KvDMR*, or in some cases, large segments that had regulatory activities *in vitro* (Supplementary Figure S1). This ∼3000 bp region includes elements that control the establishment of *Kcnq1ot1* imprinting, although these have not yet been delimited. Two CG islands (CGIs) downstream of the promoter are methylated on the maternal chromosome. This constitutes a primary imprinting mark inherited from the oocyte that inhibits *Kcnq1ot1* expression from that allele, allowing maternal expression of the neighboring upstream genes and the sense *Kcnq1* gene (1,12,13). In addition, the *KvDMR* contains other potential regulatory elements, including a sequence with enhancer activity *in vitro* and two *CTCF* binding sites with unknown function (6,14,15).

To refine our understanding of the endogenous roles of the sequence elements in the *KvDMR*, we generated a mutant mouse in which a region previously defined as the *Kcnq1ot1* minimal promoter (MP) *in vitro* (16) was ablated (designated as PO, or promoter-out allele). In contrast to previous studies, this deletion leaves intact the two *CTCF* binding sites as well as the two CGI (Supplementary Figure S1). Our data show that, in spite of the absence of the MP, alternative transcripts continued to emerge from alternative sites in the *Kcnq1ot1* region. Surprisingly, although the deletion did not ablate *Kcnq1ot1* expression, *Cdkn1c* was no longer monoallelic, suggesting that the residual *Kcnq1ot1* transcripts had lost silencing capability or that imprinted *Cdkn1c* expression required the MP sequence itself.

Unexpected results from our studies raised the question of whether *Kcnq1ot1* is a single entity or rather a series of overlapping transcripts. Complete elucidation of the transcript structure is required to understand the silencing mechanism attributed to it. Estimates of the length of the *Kcnq1ot1* transcript came from reverse transcriptase-polymerase chain reaction (RT-PCR) scans and RNA-sequencing (17), but these technologies could not establish that *Kcnq1ot1* is a single RNA molecule of 92 kb. By integrating publicly available conservation, strand-specific RNA-sequencing and chromatin immunoprecipitation (ChIP)-sequencing data from the *Kcnq1ot1* region, we observed many discrete sequences that exhibited features of enhancers or promoters. We reasoned that if there are multiple independent RNAs emerging from the *Kcnq1ot1* region, instead of (or in addition to) a 92 kb entity, the transcriptional start sites (TSS) could be identified by existing technologies. By performing targeted 5′ RACE and 3C assays, we show that several such regions are active transcriptionally and at least one is in contact with the *Kcnq1* promoter in the heart. Our results completely change previous assumptions on *Kcnq1ot1* and lead us to propose that the ncRNA is a by-product of the transcriptional process, which maintains an open chromatin structure allowing access to a variety of enhancers encoded in introns 10 and 11 of *Kcnq1*. Our experiments also show that deletion of a 200-bp region leads to loss of imprinting of *Cdkn1c*, strongly suggesting that this sequence is a long-distance silencer. These studies are valuable for understanding the mechanisms that govern imprinting in the context of other co-existing regulatory mechanisms.

## MATERIALS AND METHODS

### Targeting of embryonic stem cells

Targeting strategy is illustrated in Supplementary Figure S2A. pTV-*Kcnq1ot1-MP* was constructed by Genebridges (Heidelberg, Germany) and modified to add the negative selection marker DTA (*Diphtheria toxin A fragment*) (Taconic, Germantown, NY, USA). The targeting vector was linearized with SalI and electroporated into B6-3 ES cells. G418-resistant clones were isolated and restriction enzyme digestions and Southern blots were used to confirm accurate homologous recombination (Supplementary Figure S2B). Two confirmed targeted clones underwent a second round of electroporation with Flp to delete the neomycin-resistance (*neo*R) marker, and G418-sensitive clones were identified and confirmed with long-range PCR (Supplementary Figure S2C). Two confirmed *neo*R-deleted clones were injected into BALB/c mouse blastocysts and chimeric mice were generated.

### Mice

All mouse protocols were approved by the Institutional Animal Care and Use Committee at Temple University Medical School. For each chimeric line, germline transmission of the targeted mutation was confirmed by PCR genotyping of progeny from chimeric mice crossed with C57BL/6 mice on DNA isolated from tail biopsies (see Supplementary Table S1 for primer sequences and Supplementary Figure S2 for schematic and results of genotyping PCR). Nomenclature indicates the maternal allele first. The *Kcnq1ot1* MP was excised by crossing heterozygous mutant mice (*Kcnq1ot1^+/^*^flox-MP^) to mice expressing CMV-*Cre* recombinase (obtained from Jackson Lab, Bar Harbor, Maine, USA). Excision of the MP was confirmed in the progeny by PCR analysis (Supplementary Figure S2D). Positive heterozygous progeny, designated as *Kcnq1ot1^+/PO^*, were maintained by backcrossing to C57BL/6 or bred to homozygosity.

To distinguish the parental alleles for allelic assays (for RNA and methylation analyses, see below), mutant mice were crossed to C57BL/6(CAST7), a consomic strain homozygous for a *Mus musculus castaneus* chromosome 7 (18). In all experiments, mutant mice were compared to their wild-type (WT) littermates. All results were confirmed in two independent mutant lines.

To obtain embryonic tissues, reciprocal crosses were set up between C57BL/6 and C57BL/6(CAST7) mice, or between *Kcnq1ot1^+/PO^* and C57BL/6(CAST7) mice. Noon of the day of the plug was designated E0.5. Pregnant dams were killed at the appropriate day of gestation and fetal tissues were dissected and frozen for further analysis. Neonatal tissues were obtained from postnatal day 2 pups.

### Gene-specific-primed cDNA synthesis

RNA was extracted from snap frozen 10.5 dpc, 12.5 dpc, neonatal and adult tissues using the Roche High Pure RNA Kit (Nutley, NJ, USA). An additional DNase step was performed using Invitrogen Turbo DNase (Grand Island, NY, USA). GSP cDNA was generated for each specific qPCR analysis using Invitrogen SuperScript III at 65°C incubation for 50 min, with controls including minus reverse transcriptase and minus primers indicating no contaminating DNA nor non-specific cDNA synthesis. Samples were either used immediately for qPCR or frozen at −20°C. Primers are listed in Supplementary Table S1.

### Quantitative RT-qPCR

Immediately following GSP cDNA synthesis, RT-qPCR was performed using 1x Power Sybr Green (Invitrogen) according to manufacturer's protocol with primer-optimized reaction conditions (primers and efficiencies listed in Supplementary Table S1) and according to MIQE guidelines. PCR products were analyzed both by melt curve analysis and polyacrylamide gel electrophoresis. Mean Ct values for each triplicate reaction were normalized to geometric mean of expression values of *β-actin*, *Ppia* and *Rplp1*, three housekeeping genes that show no expression variation across cardiac developmental stages nor between heart and brain. Statistical analyses were performed using Student's *t*-test with GraphPad Prism software v6. Results are the average of three independent biological replicates (one mouse from each of three different litters), each performed in triplicate.

### 5′ and 3′ rapid amplification of cDNA

5′ and 3′ rapid amplification of cDNA ends (RACE) was performed with the 5′ and 3′ RACE systems (Invitrogen). For 5′ RACE, primers were located downstream of the predicted transcription factor binding sites. For 3′ RACE, primers were anchored at 100, 500 and 1000 bp away from the TSS found by 5′RACE. PCR products amplified according to the manufacturer's recommendation were run on 3% agarose gels and bands were extracted, cloned and sequenced. Sequences were aligned with BLAT in the UCSC genome browser. Primers for 5′ and 3′ RACE assays are listed in Supplementary Table S1.

### Protein expression analysis

For western blot analyses, total proteins were isolated from mouse neonatal hearts using RIPA buffer [20 mM Tris (pH 8.0), 150  mM NaCl, 0.1% sodium dodecyl sulfate (SDS), 1% NP-40, 0.5% sodium deoxycholate]. Protein samples (30 μg) were separated on 10% SDS–polyacrylamide gels and subjected to a standard western procedure. Antibodies for Kcnq1 and β-actin were purchased from Santa Cruz (SC-20816 and SC-47778, respectively), and both were used at a 1:400 dilution. Secondary antibody was horseradish peroxidase conjugated and used at a 1/5000 dilution. Blot was visualized on an Alpha Innotech imaging system and quantified with the Alpha FluorChem software.

### Chromatin immunoprecipitation

Tissues were extracted from postnatal day 2 (P2) mice and cross-linked in 0.5% formaldehyde in phosphate buffered saline (PBS) and protease inhibitors for 5 min (heart) or 1% formaldehyde for 10 min (brain) and quenched with 0.125 M glycine. Intact nuclei were isolated from tissue digestions and split so that each subsequent reaction had 5 × 10^6^ nuclei. Micrococcal nuclease digestion in 1 mM CaCl_2_ was performed [20 U for 1 min] and the reaction was terminated with 2 mM ethylene glycol tetraacetic acid (EGTA), followed immediately by sonication in a 0.1% SDS–PBS solution. Direct ChIP was performed on the soluble nuclear extract at 4°C overnight using either *CTCF* antibody (Active Motif, Carlsbad, CA, USA) or IgG control antibody (Vector Laboratories, Burlingame, CA, USA). Substrates were pulled down with Invitrogen Protein A Dynabeads. After washing and elution, samples were incubated with Proteinase K overnight at 65°C to reverse cross-links and enriched DNA was purified with phenol–chloroform. Ethanol precipitated DNA was resuspended in 10 mM Tris–HCl solution and analyzed by qPCR using the ActiveMotif qPCR Analysis Kit. Each ChIP assay was performed three times and qPCR results represent the mean ± SEM. ChIP primers are listed in Supplementary Table S1.

### Bisulfite mutagenesis sequencing

1 ug of DNA was mutagenized using the EZ-DNA Methylation Gold Kit (Zymo Research #5005) following the manufacturer's protocol. Amplification was performed with nested primers (listed in Supplementary Table S1) and PCR products cloned into the pCR2.1-TOPO vector using a TOPO-TA Cloning Kit (Life Technologies#K4500-01). The bacterial colonies were cultured and plasmids purified using a Spin Mini Prep Kit (QIAGEN #30020). Sequencing of the plasmid inserts was done by Eurofins MWG Operon. Sequences were analyzed with Geneious Pro 6.5. Data were obtained from three independent biological samples, with at least 10 clones sequenced from each.

### Pyrosequencing

Biotin-labeled PCR products were amplified from either cDNA or bisulfite-treated genomic DNA using Qiagen PyroMark Custom Assay Primers and Hot Start Taq Polymerase (Qiagen Catalogue #203203). The list of PCR primers and sequencing primers is provided in Supplementary Table S1. Pyrosequencing was carried out in duplicate on a PSQ96 system with a Pyro-Gold reagent Kit (Qiagen Catalogue #972804) and the results were analyzed by PyroMark Q96 ID software version 1.0 (Qiagen). Known polymorphisms (http://phenome.jax.org/) between *M. musculus castaneus* and C57BL/6 genomes were used to determine maternal/paternal contributions with Allele Quantification (AQ) assays. For methylation assays, the ratio of C-to-T at cytosine residues within CpG islands was quantified.

### Methylated DNA immunoprecipitation (MeDIP)

Genomic DNA was extracted from snap-frozen hybrid neonatal (P2) tissues using the Quick-gDNA MiniPrep Kit (Zymo Research #D3024). Using the EpiSeeker MeDIP Kit (Abcam #ab117133), 1 ug of the extracted gDNA was then subject to sonication and immunoprecipitation according to manufacturer's protocol. PCR was performed on the methylated DNA enriched samples alongside *IgG* and *Input* controls with the Hot Start Taq Polymerase Kit (Qiagen #203203) and with PyroMark Custom Assay Primers (listed in Supplementary Table S1). The resulting biotinylated PCR products were bound to Strepavidin Sepharose beads (GE Healthcare #17-5113-01) and pyrosequenced in duplicated on the PSQ96 Pyrosequencing Platform with the Pyro-Gold reagent Kit (Qiagen #972804). Resulting Allele Quantification (AQ) data were analyzed by PyroMark Q96 ID software version 1.0 (Qiagen) and percent maternal/paternal contribution was determined by comparing known polymorphisms between *M. musculus castaneus* and C57BL/6 mouse strains. Successful enrichment of methylated DNA was confirmed by comparing AQ data from immunoprecipitated samples against comparable *IgG* and *Input* controls.

### Transgenic embryo assays

A 500-bp region spanning 5 sequences, including the *Tbx5* candidate binding site was amplified from C57BL/6J genomic DNA and subcloned into the *Hsp68*-*Lac*Z vector. For microinjection, constructs were linearized, gel purified by electroelution and run through the Wizard DNA Clean Up columns (Promega). DNA was injected into male pronuclei of C57BL/6J embryos, and pseudopregnant Swiss Webster females (Taconic) were used as recipients. Transgenic embryos were collected at 11.5 dpc and stained with X-gal (Sigma) for 20 min to detect β-galactosidase activity. Images were obtained with an Olympus MVX10 stereoscope, cropped and levels adjusted with Adobe Photoshop.

### Chromosome conformation capture (3C)

Chromosome conformation capture (3C) assays were conducted as previously reported (19) with the appropriate controls (20). Cross-linked chromatin substrates were digested with AflIII and NcoI. Purified DNA samples were analyzed on 0.8% agarose gels to estimate concentration. The linear range of amplification was determined for each sample by serial dilution. An appropriate amount of DNA within the linear range (typically 500 ng of DNA) was subsequently used for the experiments. The control template was prepared from BACs spanning the *Kcnq1, Kcnq1ot1* and *Cdkn1c* genes. The linear range of the control template was determined with a serial dilution of the random ligation mix made in the same amount (500 ng) of digested and ligated BAC DNA. Primer sequences are listed in Supplementary Table S1. All PCR products were cross-linking and ligation-dependent. PCR products were run on 7% polyacrylamide gels. Intensity of the fragments was quantified on a Typhoon 9200 imager (Molecular Dynamics) and normalized between gels. All data points were generated from three different biological replicates, each performed in triplicate. Relative cross-linking frequency for a fragment X was calculated as: *γ*(*x*) = [*I*(A-X)^3C^/*I*(A-X)^C^]/[*I*(A-NF)^3C^/*I* (A-NF)^C^, where *I*(A-X)^3C^ is the intensity of PCR products from ligations between the anchor (A) and another fragment in the *Kcnq1ot1* region in the 3C substrate; *I*(A-X)^C^ is the same for the BAC control template; *I*(A-NF)^3C^ is the intensity of PCR products from ligations between the anchor and the nearest fragment to the anchor in the 3C substrate; *I*(A-NF) is the same for the BAC control template. Data were expressed as mean ± SD.

### Bioinformatics analysis

RNA-seq data were downloaded from the Genome Browser and imported into the Integrative Genomics Viewer (IGV) (21,22). Annotation for Mouse Genome (mm9) was selected and the RefSeq gene table visualized. After choosing the correct region (*Kcnq1ot1*), spliced reads from RNA-seq data were used to predict putative spliced isoforms and a Sashimi plot was generated with the Sashimi tool in the Integrative Genomics viewer (22).

## RESULTS

### Generation of the *Kcnq1ot1^PO^* allele

To determine the effect of deleting the previously defined MP for *Kcnq1ot1*, mice carrying a mutant allele at the endogenous locus were generated by homologous recombination in embryonic stem (ES) cells (Supplementary Figure S2). This allele, designated as *Kcnq1ot1^PO^* (Promoter-Out), removes 564 bp of sequence and leaves two *CTCF* sites and two CG-islands (CGIs) intact. A comparison of the *Kcnq1ot1^PO^* allele with previously generated deletions is shown in Supplementary Figure S1, illustrating that *CTCF* sites were either eliminated or partially truncated in those studies. Mutant progeny inheriting the deletion maternally or paternally were assayed for defects in *Kcnq1ot1, Cdkn1c* and *Kcnq1* expression and imprinting, *CTCF* binding and alterations in chromosome conformation. Parental origin of the alleles was distinguished by crossing heterozygous mutant mice with B6(CAST7) mice, a consomic strain in which chromosome 7 is derived from *M. musculus castaneus* (23).

### *CTCF* exhibits biallelic binding to the *KvDMR* in the neonatal heart in both wild-type and *Kcnq1ot^PO^* mice

**Figure 2.**
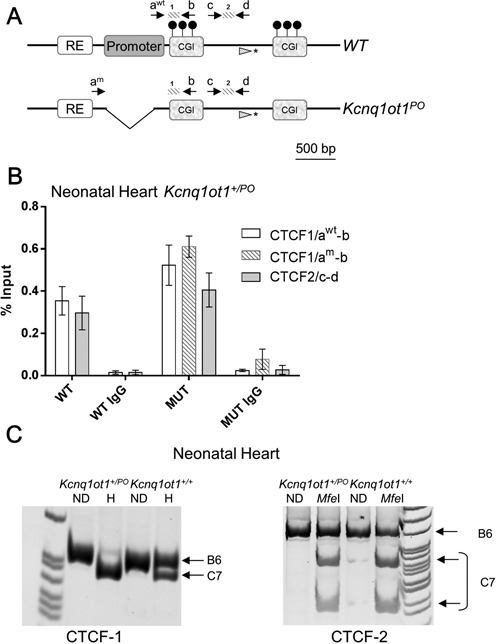
Chromatin immunoprecipitation analysis for CTCF at the *KvDMR.* (**A**) Schematic of the wild-type (WT) and *Kcnq1ot1^PO^* alleles. Primers **a^wt^** and **b** amplify CTCF binding site 1 (*CTCF*-*1*) in the WT allele only, since a^wt^ anneals to a sequence in the promoter, which is absent in the mutant allele; primers a^m^ and b amplify CTCF binding site 1 in the mutant allele (PO). Primers **c** and **d** amplify CTCF binding site 2 (*CTCF*-2) of both the WT and *Kcnq1ot^PO^* allele. Asterisk denotes SNP analyzed with the primer depicted with the hatched arrowhead. (**B**) Quantitative PCR of immunoprecipitated substrates from neonatal hearts of WT mice and *Kcnq1ot1^+/PO^* littermates. Assays were performed on mice from three litters each, in triplicate, and results expressed as percentage input normalized to H19 R1 (repeat 1 of the differentially methylated domain). Error bars indicate SEM. CTCF-1 shows occupancy of *CTCF* in both parental alleles of in both the WT mouse and the *Kcnq1ot1^+/PO^* mouse. For the *Kcnq1ot1^+/PO^* mouse (MUT), the white bar represents the data for the WT maternal (primers a^wt^ and b) and the hatched bar represents the mutant paternal allele (primers a^m^ and b) *CTCF-2* shows occupancy of *CTCF* for both WT and mutant alleles. (**C**) Allele-specific restriction digests of ChIP-PCR products for primer pairs for *CTCF*-1 (left panel) and *CTCF-*2 (right panel). ND, non-digested; H, Hpy1881 digest. For *CTCF-1*, Hpy1881 cuts only the *castaneus* allele; for *CTCF*-2, MfeI cuts only the *castaneus* allele.

Two *CTCF* sites located immediately downstream of the MP have been proposed to play a role in *Kcnq1ot1* expression or as insulators regulating access of neighboring genes to tissue-specific enhancers (24) (Figure 2A). Allele-specific binding of *CTCF* to the unmethylated paternal sites has also been reported, suggesting a role in establishing or maintaining differential methylation at the *KvDMR*. We performed ChIP assays using anti-*CTCF* antibodies on neonatal hearts of WT mice. Quantitative PCR with primers for *CTCF* binding sites 1 and 2 (*CTCF-*1, *CTCF*-2) showed positive occupancy of *CTCF* (Figure 2B, WT). To determine allele-specificity of binding, we performed allele-specific restriction digests of the PCR products from immunoprecipitated DNA. Surprisingly, we observed biallelic *CTCF* binding at the *KvDMR* in the heart (Figure 2C).

To determine if deletion of the MP affected *CTCF* occupancy, ChIP was performed with chromatin purified from neonatal hearts of *Kcnq1ot1*^+/*PO*^ mice (Figure 2B, MUT). Quantitative PCR with the primers specific for the WT (maternal) and mutant (paternal) alleles for binding site 1 yielded a positive signal for both. To confirm that *CTCF* was occupying the maternal allele at binding site 1, we performed allele-specific restriction digests on the PCR product amplified with WT-specific primers. The PCR product was the expected size, and digestion revealed *CTCF* binding to the maternal B6(CAST7) allele. Primers for *CTCF*-2, which detect both WT and mutant alleles, were also positive for *CTCF* occupancy and allele-specific restriction digests of the PCR products showed binding to both alleles (Figure 2C).

To confirm this result with a different assay, we quantified allelic presence of *CTCF* by pyrosequencing immunoprecipitated substrates (primers and the polymorphism analyzed indicated in Figure 2A). Although *CTCF* occupancy was higher on the paternal allele, we observed significant enrichment at the maternal allele relative to input samples (for *Kcnq1ot1*^+/*PO*^, *P* = 0.004, *n* = 6; for *Kcnq1ot1*^+/+^ *P* < 0.0001, *n* = 4). Thus, methylation of the maternal allele does not affect *CTCF* binding to its cognate sites at the *KvDMR*. Importantly, *CTCF* binding is not perturbed by the absence of the MP, making it a good model for testing the role of *CTCF* in the *Kcnq1ot1* region.

### *Cdkn1c* loses imprinted expression in the absence of the *Kcnq1ot1* minimal promoter

**Figure 3.**
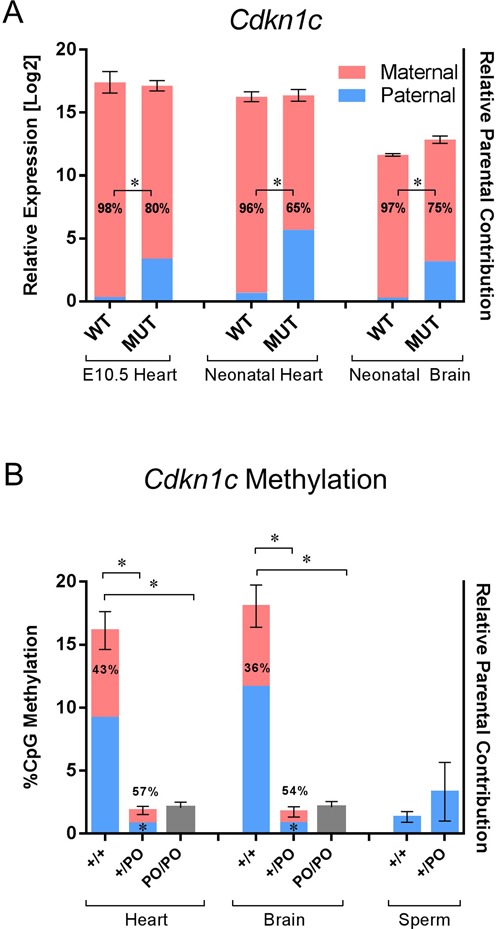
Effects of the paternal inheritance of the *Kcnq1ot1^PO^* allele on expression, imprinting and methylation of *Cdkn1c.* (**A**) Combined analysis of cardiac and brain *Cdkn1c* expression and imprinting in wild-type and *Kcnq1ot1^+/PO^* mice as assayed by qPCR and quantitative pyrosequencing, respectively. For qPCR, mean CT values were normalized to the geometric mean of *β-actin, Ppia* and *Rplp1.* Error bars indicate SEM. Numbers inside the bars are the percent maternal allele abundance relative to total *Cdkn1c* expression. Asterisks between bars indicate significant difference in parental contribution (*P* < 0.0001). (**B**) Methylation analysis of the *Cdkn1c* promoter in wild-type (+/+), *Kcnq1ot1^+/PO^* (+/PO) and *Kcnq1ot1^PO/PO^* (PO/PO) tissues by pyrosequencing. Bisulfite-treated genomic DNA was subjected to PCR with biotin-labeled primers; pyrosequencing results were analyzed for ratio of C to T transitions. Asterisks above the bars indicate a significant difference in methylation levels (*P* < 0.0001). Numbers inside the bars are percent maternal allelic abundance relative to total *Cdkn1c* expression and asterisks indicate a significant difference in parental contribution compared to wild-type (*P* = 0.002 for heart, *P* = 0.0003 for brain, *n* = 4).

To determine the effect of deleting the *Kcnq1ot1* MP on imprinted expression of *Cdkn1c*, we performed quantitative pyrosequencing of expressed strain-specific single nucleotide polymorphisms (SNPs). Although *Cdkn1c* was monoallelic in all WT tissues, *Kcnq1ot1^+/PO^* mice showed biallelic expression of *Cdkn1c* in every tissue tested (Figure 3A). Paternal expression of *Cdkn1c* was significantly higher in *Kcnq1ot1^+/PO^* than in WT mice (for all stages, *P* < 0.0001), indicating that absence of the MP releases the repression of the paternal *Cdkn1c* allele. However, the maternally biased expression was maintained, suggesting that repressive epigenetic marks were not completely removed. Surprisingly, despite the biallelic expression pattern, total RNA abundance of *Cdkn1c* does not increase significantly in the *Kcnq1ot1^+/PO^* heart and brain, as assayed by qPCR. This implies the existence of a feedback mechanism to limit *Cdkn1c* levels in those tissues. *Cdkn1c* maintained a monoallelic pattern in all neonatal tissues from progeny of the reciprocal cross (*Kcnq1ot1^PO/+^*) (Supplementary Figure S3).

The *Cdkn1c* gene lies in a CG-rich region that harbors a secondary methylation mark on the paternal allele (25), and it has been suggested that the *Kcnq1ot1* ncRNA is directly responsible for recruiting DNA-methyltransferases to establish this modification (26,27). We tested the methylation status of the promoter in WT, *Kcnq1ot1^+/PO^* and *Kcnq1ot1^PO/PO^* mice by bisulfite mutagenesis and pyrosequencing. The *Cdkn1c* promoter is unmethylated in sperm from WT and mutant (heterozygous or homozygous) mice. In neonatal heart and brain tissues of the *Kcnq1ot1^+/PO^* and *Kcnq1ot1^PO/PO^* mice, the *Cdkn1c* promoter exhibits complete lack of parental methylation compared to WT mice (Figure 3B). This is in agreement with the biallelic expression in the mutants, indicating that transcription from or physical presence of the *Kcnq1ot1* MP is required for acquisition of the secondary mark at the paternal *Cdkn1c* allele.

### The mono- to biallelic transition for *Kcnq1* is maintained in *Kcnq1ot1^+/PO^* cardiogenesis

**Figure 4.**
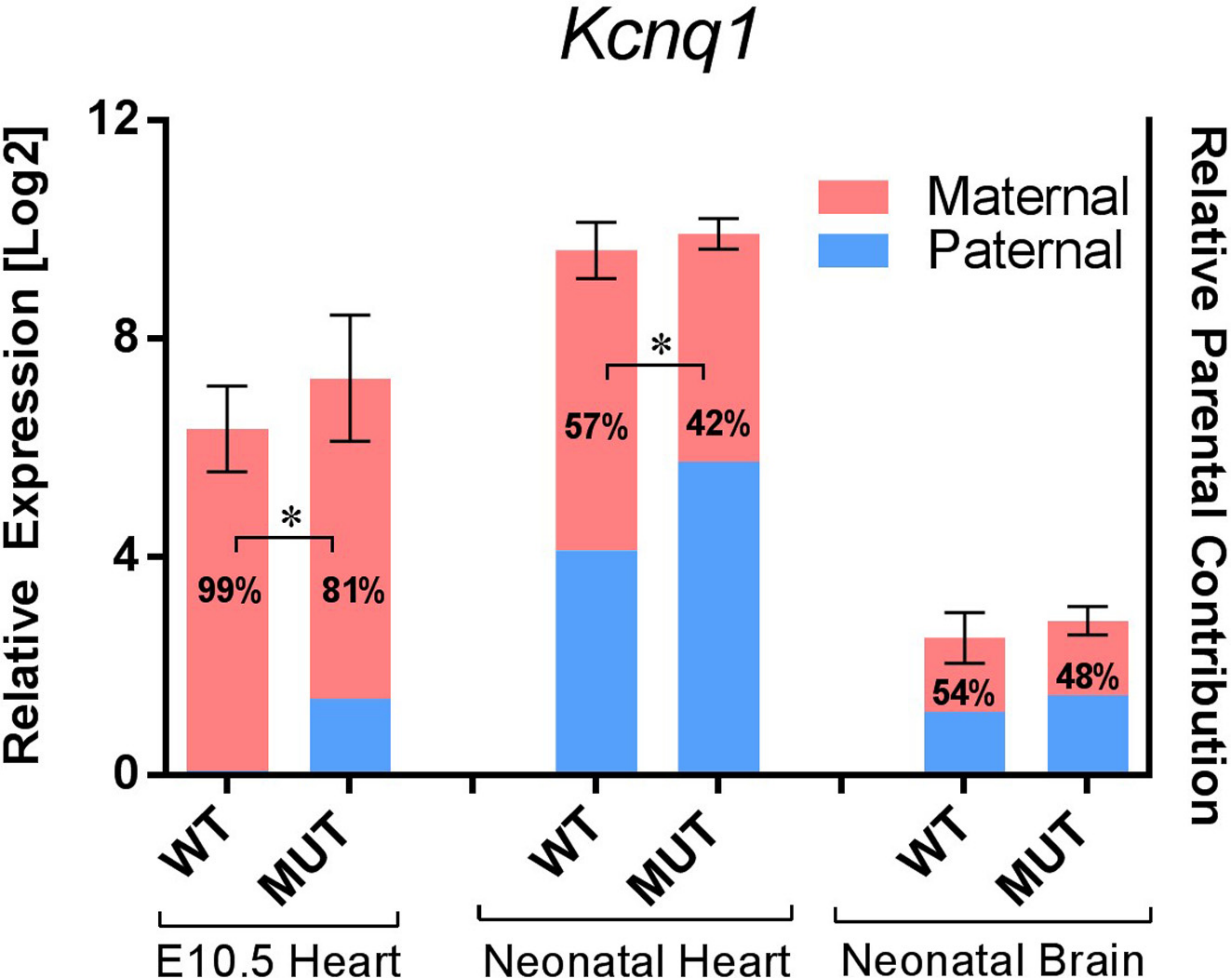
Effects of the paternal inheritance of the *Kcnq1ot1^PO^* allele on expression and imprinting *Kcnq1*. Combined analysis of cardiac and brain *Kcnq1* expression and imprinting in wild-type and *Kcnq1ot1^+/PO^* mice (assays as described in Figure 3A). Error bars indicate SEM. Numbers inside the bars are percent maternal allelic abundance relative to total *Kcnq1* expression. Asterisks between the bars indicate a significant difference in parental contribution compared to wild-type (*P* = 0.0011 for 10.5 dpc heart, *P* < 0.0001 for neonatal heart, *n* = 4).

In WT mice, *Kcnq1* transitions from mono- to biallelic mode at 13.5 dpc in all the expressing tissues (8). This pattern is disrupted in brain of the *Kcnq1ot1^+/PO^* mice, with full biallelic expression of *Kcnq1* in early stage embryos (not shown). In the fetal heart, however, absence of the MP results in only partial loss of imprinted expression, with paternal levels contributing less than 20% of the total RNA abundance in 10.5 dpc hearts, as assayed by pyrosequencing (Figure 4). A transition to full biallelic expression is still noticeable, with maternal and paternal contributions to *Kcnq1* expression equalizing in *Kcnq1ot1^+/PO^* neonatal hearts. Together with results from previous studies (9), these data underscore that developmentally regulated imprinting patterns of the *Kcnq1* region in the early stage heart are from other tissues.

To determine if the *Kcnq1ot1* MP sequence *per se* contributed to the RNA levels of *Kcnq1*, we performed RT-qPCR for *Kcnq1* from 10.5 dpc and neonatal heart and neonatal brain in *Kcnq1ot1^+/PO^* mice. Absence of the *Kcnq1ot1* MP had no effect on levels of *Kcnq1*, even though both maternal and paternal alleles were now expressed (Figure 4). Protein levels of *Kcnq1* were also invariant in in *Kcnq1ot1^+/PO^* neonatal hearts (Supplementary Figure S4), suggesting that transcription from the MP or the MP sequence itself is not required for full activation of *Kcnq1*.

### The *Kcnq1ot1* ncRNA is still expressed in the absence of the minimal promoter

**Figure 5.**
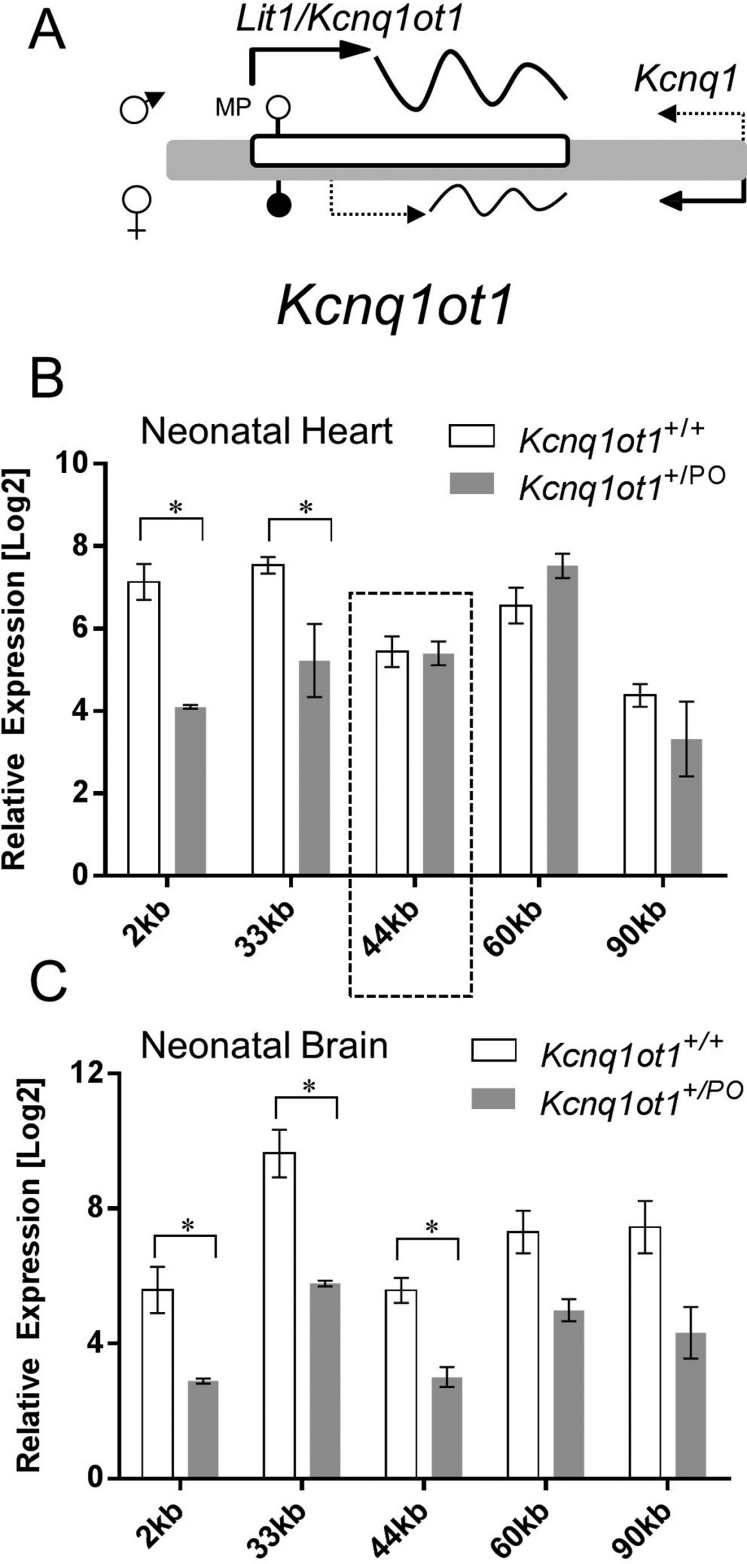
Analysis of cardiac and brain *Kcnq1ot1* ncRNA expression. (**A**) Schematic of *Kcnq1ot1* region (not to scale). Filled and unfilled circles are methylated and unmethylated CGIs, respectively and dotted arrow represents maternal transcription of the ncRNA in the heart. (**B**) Comparison of *Kcnq1ot1* levels in neonatal hearts of wild-type and *Kcnq1ot1*^+/PO^ mice with primers located 2, 33, 44, 60 and 90 kb downstream from the canonical transcriptional start site. For qPCR, mean CT values were normalized to the geometric mean of *β-actin, Ppia* and *Rplp1*. Error bars indicate SEM. Asterisks indicate significant difference between wild-type and *Kcnq1ot1*^+/PO^ (for 2 kb, *P* = 0.005, *n* = 4; for 33 kb, *P* = 0.0245, *n* = 4). The dotted box indicates the region in which expression is consistently diminished, coinciding with a candidate enhancer region (see Figure 7, main text). (C) Comparison of *Kcnq1ot1* levels in neonatal brains of wild-type and *Kcnq1ot1*^+/PO^ mice with primers located 2, 33, 44, 60 and 90 kb downstream from the canonical TSS. For qPCR, mean CT values were normalized to the geometric mean of *β-actin, Ppia* and *Rplp1*. Error bars indicate SEM. Asterisks indicate significant difference between wild-type and *Kcnq1ot1*^+/PO^ (for 2 kb, *P* = 0.0478, *n* = 4; for 33 kb, *P* = 0.0024, *n* = 4; for 44 kb, *P* = 0.0162, *n* = 4).

Loss of imprinted *Cdkn1c* expression in the *Kcnq1ot1^+/PO^* mouse could be due to absence of the *Kcnq1ot1* ncRNA or to an intrinsic regulatory activity of the MP. To distinguish between these two possibilities, we compared the levels of *Kcnq1ot1* transcript in neonatal hearts and brains of WT and *Kcnq1ot1^+/PO^* mice, by performing RT-qPCR with sets of primers that spanned the entire 92 kb length of the RNA (Figure 5). Contrary to expectations, deletion of the MP did not lead to absence of the *Kcnq1ot1* transcript in any tissue. The RNA levels in the *Kcnq1ot1^+/PO^* hearts were significantly lower with primer sets at 2 and 33 kb downstream of the canonical TSS (*P* = 0.0021 and *P* = 0.0045, respectively), but they were similar to WT levels with primer sets further downstream (44, 60 and 90 kb) (Figure 5B). On the other hand, *Kcnq1ot1* ncRNA levels in *Kcnq1ot1^+/PO^* brains showed significant decreases with primer sets 2, 33 and 44 kb (*P* = 0.0478, 0.024 and 0.0162, respectively), and a trend toward lower expression with the remaining primer sets further downstream (Figure 5C). These results show (i) that WT levels of RNA resulting from transcription throughout the *Kcnq1ot* region are not uniform; (ii) that the sequence previously defined as the MP is not essential for transcription throughout the *Kcnq1ot1* region and (iii) that in the heart, there are tissue-specific alternative TSS more than 33 kb downstream of the MP that act independently of it.

To determine the imprinting status of *Kcnq1ot1* in *Kcnq1ot1^+/PO^* neonatal hearts and brains compared to WT, and to test whether parental contributions were similar throughout the locus, we performed pyrosequencing analysis at 2, 33, 44, 60 and 90 kb downstream of the MP. In the heart, loss of imprinting of *Kcnq1ot1* was not observed at 2 kb, but was evident with primers from 33 kb onward. This was true in both in both WT and *Kcnq1ot1^+/PO^* mice in the cardiac tissue (Supplementary Figure S5B and SC). In contrast, paternal-specific expression was maintained in brain independently of the presence or absence of the MP (Supplementary Figure S5D and SE). Thus, the loss of imprinting of *Kcnq1ot* is a tissue-specific phenomenon. Activation of maternal allele suggests the presence of alternative cardiac promoters not subject to the imprinted *KvDMR*.

We quantified the contributions of the maternal and paternal alleles throughout the *Kcnq1ot1* locus in neonatal hearts. In the WT mouse (Supplementary Figure S5B), a significantly greater contribution of maternal expression is seen with primers located at 44 kb downstream of the MP, relative to that observed at 2 kb (*P* < 0.0001). In the *Kcnq1ot1^+/PO^* hearts (Supplementary Figure S5C), all primer sets showed a higher maternal proportion of total expression relative to 2 kb (for 33 kb, *P* = 0.0018; for 44, 60 and 90 kb, *P* < 0.0001).

In the WT heart, maternal *Kcnq1ot1* transcripts emerge from alternative start sites (TSS) that bypass the methylated CGIs downstream of the MP. We performed 5′ RACE assays to determine if these sites were active on the maternal allele in *Kcnq1ot1^+/PO^* neonatal hearts. Our results confirm that the maternal-specific TSS are activated normally in the mutant mice (Supplementary Figure S6), suggesting that the maternal contributions to *Kcnq1ot* expression are not captured equally with all primer sets, but depend on their location relative to the alternative TSS.

### The minimal promoter is required for maintenance but not establishment of the hypomethylated paternal *KvDMR*

**Figure 6.**
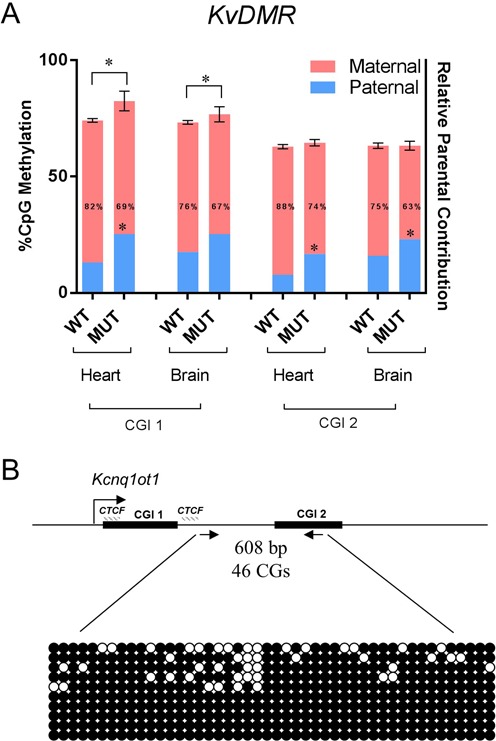
Methylation analyses of the *KvDMR* in the absence of the *Kcnq1ot* minimal promoter. (**A**) Combined analysis of percent total methylation (as assayed by pyrosequencing) and allelic distribution of the methylation (as assayed by MeDIP) between *Kcnq1ot^+/+^* and *Kcnq1ot1^−/PO^* neonatal heart and brain at the two CG islands downstream of the *Kcnq1ot1* promoter (CGI 1 and 2). Asterisks above the bars indicate a significant difference in methylation levels (*P* = 0.008 for heart, *P* = 0.0017 for brain at CG1). Numbers inside the bars are percent maternal methylation relative to total and asterisks indicate a significant difference compared to wild-type (*P* = 0.0012 for heart at CGI 1, *P* < 0.0001 for heart, *P* = 0.0005 for brain at CGI 2, *n* = 4). (B) Bisulfite mutagenesis sequencing of oocytes from *Kcnq1ot1^−/−^* females. Arrows indicate the primers that amplify a 608 bp sequence including CGI 2, with a total of 46 CGs (primers in Supplementary Table S1). Top, schematic of the amplified region, indicating the CGIs, the *CTCF* binding sites and the direction of transcription of *Kcnq1ot*. Below, representation of the methylation status of 10 individual DNA strands. Filled circles, methylated cytosines.

Figure 1 shows the *KvDMR*, a CG-rich region that is hypermethylated maternally and methylation-free paternally. To determine if the MP is required to prevent establishment of paternal methylation, we assayed DNA methylation by pyrosequencing in *Kcnq1ot1^PO/PO^* sperm and *Kcnq1ot1^+/PO^* neonatal heart and brain at the two CG islands (CGI 1 and 2) (Figure 6A). Although the *KvDMR* in sperm from mutant mice completely lacked methylation (not shown), the absence of the MP led to an increase in methylation of the region in neonatal tissues. The higher methylation levels were contributed by an increase in methylation of the paternal allele. Thus, the region remains refractive to *de novo* but not maintenance DNA methylation.

To determine if, on the other hand, the MP is required for normal acquisition of maternal methylation, bisulfite mutagenesis sequencing was performed at the *KvDMR* in *Kcnq1ot1^PO/PO^* oocytes (Figure 6B). Methylation was acquired normally in the *KvDMR* region, indicating that the minimal *Kcnq1ot1* promoter is not required for DNA methylation establishment during oogenesis.

### *Kcnq1ot1* levels are inversely correlated with *Kcnq1* expression in many tissues

**Figure 7.**
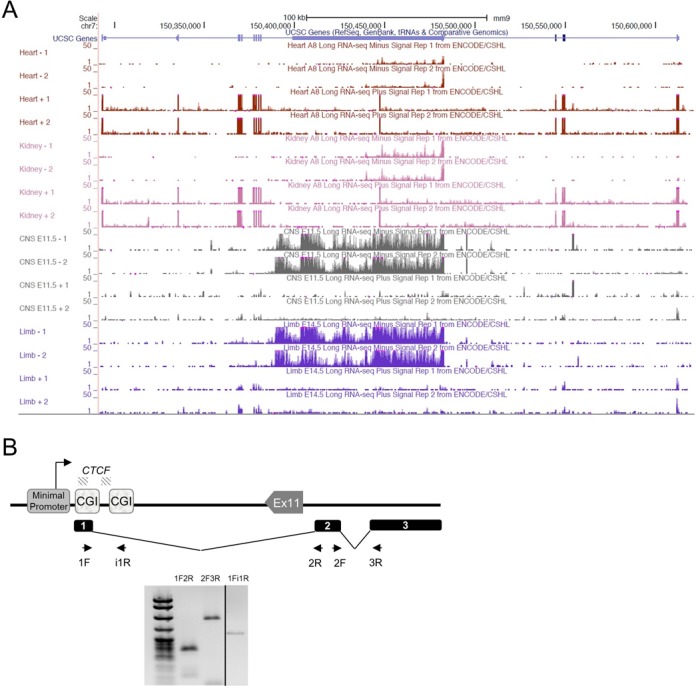
The *Kcnq1ot1* ncRNA is spliced. (**A**) Long RNA-sequencing data showing strand-specific expression from ENCODE, visualized in the UCSC genome browser. Minus strand codes for the *Kcnq1ot1* lncRNA; the plus strand codes for *Kcnq1*. Higher *Kcnq1* expression correlates with lower and more discontinuous *Kcnq1ot1* expression. (**B**) Confirmation of predicted splicing events for the *Kcnq1ot1* lncRNA. Top, schematic of the *Kcnq1ot* coding region and the splicing variants (not to scale). cDNA from neonatal heart was used as substrates for qPCR. Shown is a 2% agarose gel with PCR products from neonatal heart. Primer sets 1F-2R and 2F-3R are designed to detect the junctions between exon 1 and 2 (190 bp), and 2 and 3 (369 bp), respectively. Primers 1F-i1R detect the unspliced version (297 bp). Exons 1 and 2 are 247 and 1043 bp, respectively.

*Kcnq1ot1* exhibits tissue-specific transcriptional regulation, with down-regulation in the heart, but maintains high levels in the brain. Because *Kcnq1*, the sense gene, is highly expressed in heart but not easily detectable in whole brain, we hypothesized that *Kcnq1ot1* levels might be inversely correlated to those of *Kcnq1*. Supporting this, previous results in which a major form of *Kcnq1ot1* is prematurely terminated showed that *Kcnq1* exhibited increased RNA levels in the heart (19). We examined strand-specific RNA-sequencing data in a range of tissues available from the ENCODE consortium; this confirmed that *Kcnq1ot1* levels are inversely correlated with *Kcnq1* in many tissues, suggesting that their expression is mutually exclusive (28) (Figure 7A). We also observed a trend toward higher expression of *Kcnq1ot1* in embryonic compared to adult tissues.

### The *Kcnq1ot1* ncRNA is not a single entity

Several mechanisms have been proposed for regulation by antisense transcription, including direct effects of the non-coding RNAs (26) and/or the act of transcription itself as a source of transcriptional interference (29). To explore these possibilities, we turned our attention to fully characterizing the *Kcnq1ot1* transcript.

When scanning the abundance of the *Kcnq1ot1* transcript with five primer sets, we noticed that RNA levels were unequal, with a drop in abundance at 44 kb downstream of the major TSS that was reproducible in all our WT heart samples (Figure 5B). A consistent drop in RNA levels across a locus can be due to a splicing event or an alternative TSS. We explored both these possibilities.

We downloaded long strand-specific RNA-sequencing data from ENCODE/CSHL and used Sashimi plots to explore if there were alternatively spliced regions in the *Kcnq1ot1* molecule. Two possible splicing events were detected by this analysis (Supplementary Figure S7). To validate them, we designed primers spanning the predicted introns and sequenced the PCR products. Reverse transcription and PCR of liver, brain and heart RNAs produced expected product sizes, and sequencing confirmed the presence of three exons (Figure 7B).

**Figure 8.**
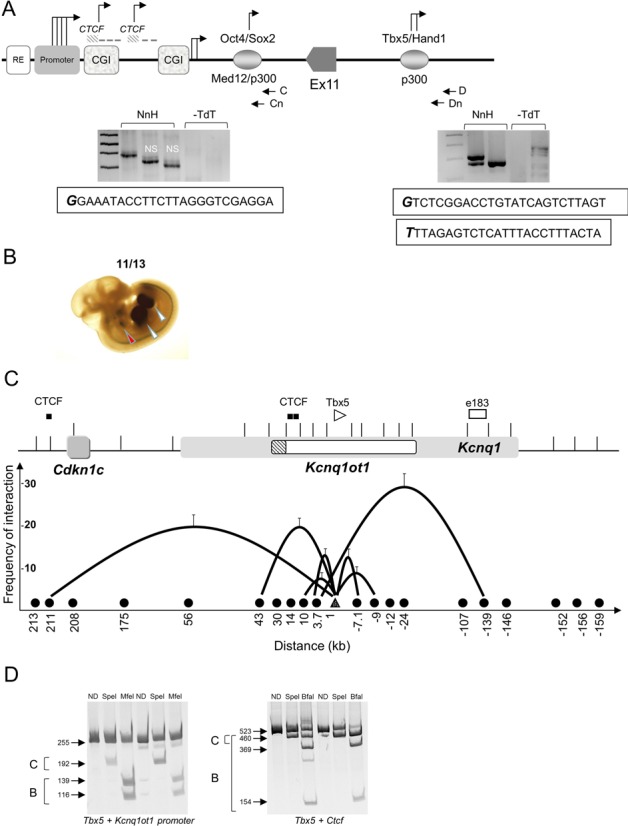
A novel heart enhancer activates *Kcnq1*. (**A**) Schematic showing the *Kcnq1ot1* MP and part of the coding region, including the *Oct4* and *Tbx5* binding sites (not to scale). The *Oct4* site is 7 kb downstream of the MP and the *Tbx5* site is 10 kb downstream of Exon 11 and 45 kb downstream of the MP. 5′ RACE analyses were performed with nested primers, C and Cn for *Oct4* site, and D and Dn for *Tbx5* site. Below, gels showing the fragments obtained and the first 25 bases of the RNA transcribed from each site. For *Oct4*, the positive fragment was 326 bp; NS, non-specific; NnH, neonatal heart; -dTd, minus terminal deoxynucleotidyl transferase. For *Tbx5*, the positive fragments were 282 and 247 bp. (**B**) 11.5 dpc transgenic embryo analysis with the *Tbx5* element. Representative LacZ-stained embryo exhibiting *in vivo* tissue-specific enhancer activity. Red arrowheads indicate the heart; white arrowheads point to the limb buds. Numbers show the reproducibility of LacZ reporter staining (11 of 13 total embryos). (**C**) Chromatin loop formation by 3C in wild-type neonatal heart. Top, schematic of the *Kcnq1* region, with the *Kcnq1ot1* promoter as a hatched box. *CTCF* sites and enhancer element 183 indicated. Bottom, graphic representation of 3C results. Black circles on the X-axis indicate sites tested; interactions between *Tbx5* binding site (white triangle) and other restriction fragments depicted by black lines, with height of the curve determined by cross-linking frequency and the X-axis representing the distance from the anchor fragment in kilobases. Error bars represent SD. (**D**) Allele-specificity of the chromatin loops in the neonatal heart. Restriction digests of the 3C products between the *Tbx5* binding site and the *Kcnq1ot1* promoter and *CTCF* site. Digestion of the 255 bp *Tbx5*–*Kcnq1ot* promoter loop with SpeI cuts the *castaneus* allele into two fragments of 192 and 63 bp (the latter not shown); MfeI digests the C57BL/6 allele into fragments of 139 and 116 bp. Digestion of the 523 bp *Tbx5*–*CTCF* loop with SpeI cuts the *castaneus* allele into 460 and 63 bp fragments (the latter not shown); BfaI digests the *castaneus* allele into fragments of 458 and 65 bp and the C57BL/6 allele into fragments of 369 and 154 bp. ND, not digested; B, C57BL/6 and C, *castaneus*.

An alternative (though not exclusive) hypothesis to explain the variable levels of the *Kcnq1ot1* transcript across the locus is that there are additional TSS contributing alternative RNAs to the total expression in the region. Several regions within introns 10 and 11 of *Kcnq1* exhibit epigenetic features consistent with enhancer or promoter activity, harbor bioinformatically predicted transcription factor binding motifs (30) and/or exhibit transcription factor binding by ChIP-sequencing. We performed targeted 5′ RACE on several of these sites to determine if they were active in neonatal heart and brain (Figure 8A). Some candidate sites did not yield results in the 5′ RACE assays, at least for heart and brain RNA. However, assays for two of the candidate regulatory regions, positive for binding of *Tbx5* in cultured heart cells (31) and *Oct4* in mouse ES cells (32), respectively, indicated that there were novel RNA species emanating from them in both neonatal heart and brain (Figure 8A). To test whether these RNAs were enhancer RNAs, we performed 3′ RACE with primers located 500, 1000 and 1500 bp from the 5′ end; no products were detected, suggesting that the RNAs produced are longer than 1500 bp or are not polyadenylated.

Thus, the *Kcnq1ot1* region produces multiple transcripts of varying sizes, emerging from a previously defined MP and at least two additional TSS. Transcripts produced from the MP can undergo splicing, with at least two introns removed from the ncRNA.

### An enhancer in the *Kcnq1ot1* region directs expression to the heart and limb buds

Enhancers can direct the production of ncRNAs that are required for activation of their corresponding promoters (33). We reasoned that RNAs emanating from the *Tbx5* and *Oct4* sites could be indicative of enhancer activity. We performed transgenic mouse assays with the *Tbx5* region. At E11.5, the *Tbx5* sequence exhibited positive β-galactosidase staining in heart and limb buds, consistent with the known regulatory activities of *Tbx5* (34–36) (Figure 8B, Supplementary Figure S8).

To determine whether the *Tbx5* binding site makes contact with the *Kcnq1ot1, Kcnq1* or *Cdkn1c* promoters to regulate their expression, we used 3C assays, which identify DNA sequences that interact *in vivo.* Cross-linked chromatin from WT neonatal hearts was analyzed by semi-quantitative PCR using an invariant primer anchor at the *Tbx5* sequence. As shown in Figure 8C, the *Tbx5* site loops to a previously identified heart enhancer (‘element 183′) (37) in intron 1 of *Kcnq1*, that in turn was shown to interact with the *Kcnq1* promoter in the heart (9). This interaction is already present in the heart at E12.5, and is absent in the brain. The *Tbx5* fragment also contacts the *Kcnq1ot1* promoter and a *CTCF* binding site 211 kb upstream that lies between the *Cdkn1c* and *Slc22a1* genes. Allele-specific restriction digests of the 3C products (Figure 8D) and sequencing of the ligated fragments indicates that the *Tbx5* loops occur on both maternal and paternal alleles.

## DISCUSSION

In this report, we provide strong evidence that DNA sequence elements and their relative location in the *Kcnq1ot1* region are responsible for imprinted expression of *Cdkn1c* and, to some degree, of early embryonic *Kcnq1*. This undermines the current paradigm whereby the *Kcnq1ot1* ncRNA is a single molecule that spreads and directly silences its neighboring genes. In the absence of the MP, a sequence that contains the main *Kcnq1ot1* promoter, *Cdkn1c* loses imprinted expression, but the ncRNA continues to be transcribed across the *Kcnq1ot1* region from multiple initiation sites. We show that *Kcnq1ot1* is not a single molecular entity, but rather encodes a complex population of overlapping RNAs, at least one of which is spliced. We propose that the transcription throughout the *Kcnq1ot1* locus in the early stage embryo serves to make regulatory elements available to their protein binding partners, setting the stage for their use later in development. In fact, we identify tissue-specific usage of a cardiac enhancer involving chromatin looping to allow physical proximity to other regulatory elements. This conformation is not present in the early embryo, suggesting it requires specific transcriptional factors to be established. We also propose that as the *Kcnq1* promoter and/or other genes contact elements within the *Kcnq1ot1* locus, alternative TSS are activated, driving expression of several overlapping ncRNAs.

### The Kcnq1ot1 minimal promoter is required for imprinting of Cdkn1c

The *KvDMR* was initially defined as a region of differential methylation between parental alleles in intron 11 of *Kcnq1*, with a ncRNA emerging from the unmethylated paternal copy in antisense direction to *Kcnq1*. Several experiments *in vivo* and *in vitro* identified a MP, an enhancer-like activity, a silencing element, two *CTCF* binding sites and several repetitive sequences (16,38,39). Targeted deletions in the *KvDMR* ranging from 200 to 4000 bp as well as insertions to engineer the premature termination of the *Kcnq1ot1* ncRNA all disrupted imprinted expression of the *Cdkn1c* gene (summarized in Supplementary Figure S1) (14,27,40), even though ncRNA transcripts continue to emerge from the region from alternative TSS that were identified later (19). Together, our data and those of other labs (7) suggest that maintenance of repression of the paternal *Cdkn1c* gene in the embryo is independent of the *Kcnq1ot1* transcript *per se*, but rather requires one or more DNA elements within the *KvDMR*.

Initial repression of *Cdkn1c* accompanied by secondary methylation at its promoter may still be dependent on the *Kcnq1ot1* transcript, as posited by several labs (27). We observed a decrease in the ncRNA abundance with primers spanning the initial 33 kb of the *Kcnq1ot1* locus in the absence of the MP. This indicates that expression of this region, though not of downstream sequence, depends on the MP. In addition, it could suggest that the first kilobases of the ncRNA have repressive activity and that below a certain threshold level, it cannot silence *Cdkn1c*.

In the WT heart, down-regulation of *Kcnq1ot1* and its loss of imprinting does not affect *Cdkn1c* imprinted expression, whereas upon removal of certain sequences within the *KvDMR*, as in the *Kcnq1ot1^+/PO^* mice, *Cdkn1c* loses imprinting in every tissue. This strongly supports the hypothesis that establishment and maintenance of *Cdkn1c* imprinting are independent events. While either the ncRNA or the act of transcription at *Kcnq1ot1* is required for the establishment of methylation at the *Cdkn1c* promoter, maintenance of monoallelic *Cdkn1c* expression thereafter depends on the continued presence of a specific DNA element localized at the *Kcnq1ot1* promoter. This is in partial agreement with previous reports ablating *Kcnq1ot1* post-transcriptionally (7).

Surprisingly, although *Cdkn1c* expression is biallelic in *Kcnq1ot1^+/PO^* mice, total RNA levels in the heart are not significantly increased. Possibly a negative feedback mechanism operates above a certain threshold of *Cdkn1c* RNA or protein. More detailed profiling of *Cdkn1c* expression in the developing heart and additional mutations are necessary to understand the regulatory mechanisms at this locus.

The effect of removing the *Kcnq1ot1* MP on *Kcnq1* imprinting is especially interesting. Full biallelic expression of *Kcnq1* is seen in the mutant mice, even at early stages which exhibit monoallelic expression in WT mice. However, in the heart, maternally biased expression is conserved and a transition to full biallelic mode is still observed after 13.5 dpc, with the same dynamics as in the WT. This suggests that the paternal *Kcnq1* allele is repressed in the heart through a unique mechanism, a question that needs to be further explored. It is interesting to note that heart-specific chromatin remodeling complexes have been identified, underscoring that proteins involved in epigenetic regulation can be diverse and tissue-specific (41). The biology of the binuclear cardiomyocytes may also require exceptional regulatory mechanisms. Single-cell technology to visualize transcriptional events should provide more detailed information on the activities in this domain. Because *Kcnq1* is an important gene for cardiac function, this region is a model for the co-existence of opposing regulatory mechanisms and how they interact, compete and prevail during development.

### Revisiting the relationship between DNA methylation, CTCF binding and transcription of DMRs

We were interested in seeing if removal of the *Kcnq1ot1* MP affected the regulatory elements within the *KvDMR* in *Kcnq1ot1^+/PO^* mice. Two main observations are particularly revealing: (i) in neonatal heart, *CTCF* exhibited binding to the maternal allele despite its hypermethylated status; (ii) the *Kcnq1ot1* region was transcribed in spite of the lack of the main promoter. These results led us to re-examine the assumptions on the structure and function of the *KvDMR* and the ncRNA itself in the WT mouse.

Previous studies reported allele-specific binding of CTCF to the *KvDMR*, and it was hypothesized that maternal methylation impeded CTCF binding, allowing *Cdkn1c* access to an upstream enhancer element (24,39). Conversely, it was suggested that CTCF occupancy of its sites on the paternal allele could have a role in protecting the region from methylation. Our results show that in the heart, CTCF is capable of occupying sites 1 and 2 (four and one CpGs, respectively) on both maternal and paternal alleles. This agrees with a report of biallelic CTCF presence at the *KvDMR* in mouse embryonic fibroblasts (42), and contrasts with previous reports, perhaps because of the differences in tissues and developmental stages. Although CTCF binding is usually assumed to be methylation-sensitive, even in the classic model of the *H19* DMD (differentially methylated domain), mutations in specific CpGs allowed binding of *CTCF* despite the presence of methylation (43). Thus, it seems that *CTCF* methylation-sensitivity may depend on the location of the CGs within its binding site and is dependent on context. Our results prove that CTCF does not function as an enhancer-blocking element at the endogenous locus, at least for *Cdkn1c*. Removal of the MP is sufficient to activate the paternal *Cdkn1c*, as noted above, despite the occupancy of CTCF on that allele. This result also suggests that the elements required for *Cdkn1c* expression in heart and brain lie downstream of the MP.

It has been hypothesized that transcription across differentially methylated regions in the oocyte is required for establishment of maternal methylation marks (44). Absence of the MP does not lead to hypomethylation when the mutation is transmitted maternally, suggesting that either that the *KvDMR* can be methylated regardless of its transcriptional status, or alternatively, that the additional TSS, if active in the oocyte, are sufficient for the methylation machinery to access the region. Alternatively, transcription across the *KvDMR* emanating from the sense *Kcnq1* in oocytes may be sufficient to afford access to the methylation machinery.

### *Kcnq1ot1* is not a single entity

Antisense transcripts often exhibit low fidelity of transcription initiation and for *Kcnq1ot1*, we had identified alternative TSS up to 1.3 kb downstream of the MP, some of which were tissue-specific (19). Surprisingly, removal of the MP did not affect activation of these sites in the *Kcnq1ot1^+/PO^* mice, with RNAs emerging from both parental alleles in the heart. This suggests that activity of alternative TSS relies on tissue-specific factors.

In our studies and in publicly available expression data, we noticed that abundance of the *Kcnq1ot1* transcript was not uniform across the locus. This was not due to difference in primer efficiencies nor unusually repetitive sequences affecting the RNA-seq data. Previous studies had not tested along the full length of the transcript, thus overlooking this uneven abundance. A possible explanation is that the *Kcnq1ot1* either has unannotated TSS or is spliced, or both. In fact, our analysis of RNA-seq data and subsequent experiments revealed two splicing events. Even more striking was the fact that there were several sites that initiated transcripts at locations that also bear features of regulatory regions. One of these harbors a motif recognized by *Tbx5*, an important factor in cardiac and limb development (34,45). *Tbx5* is part of a network of transcriptional factors that activate heart-specific genes (46). We speculated that *Tbx5* and its binding site could be important for activation of *Kcnq1* and could help determine the domain-wide conformation of the region in the heart.

### The *Kcnq1ot1* region is an enhancer jungle

Tissue-specific activation of the *Kcnq1* gene is dependent on interactions between the promoter and at least two cardiac enhancers, one of which lies within the region encoding *Kcnq1ot1*. We previously reported physical proximity between the *Kcnq1* promoter and a region in intron 1, already present in embryonic stages, with a contact established specifically in the heart at an upstream element. Here, we find an additional interaction between the intron 1 element (e183) (30) with a sequence that harbors many hallmarks of a cardiac/limb bud enhancer. Our assays to determine if these are eRNAs were inconclusive, and expression from the overlapping *Kcnq1* gene complicate the analyses. However, transgenic embryo assays revealed that one candidate, with a recognition motif for *Tbx5*, exhibits tissue-specific expression in heart and limb.

One pending question is whether *Kcnq1* expression matches the *Tbx5* pattern in the limb bud, or if this particular enhancer is used in the heart for *Kcnq1* and engages other distant genes in the limb bud. The expression domain suggested by the conformational assays includes the *Cdkn1c* gene, raising the question of how cell-cycle inhibition is coordinated with activation of cardiac-specific genes within this domain. Furthermore, it will be interesting to refine the timeline for these regulatory events, since there may be temporal differences between the maternal and paternal alleles due to their distinct chromatin status.

Overall, our data suggest that the products of the *Kcnq1ot1* region vary in molecular structure, abundance and complexity as development ensues. The ∼90 kb *Kcnq1ot1* molecule differs from other macroRNAs that act directly by silencing other genes, either by a spreading mechanism (47) or by transcriptional overlap (29). In fact, our results strongly suggest that the MP, or sequence elements within it, rather than the RNA molecule itself, are required for *Cdkn1c* repression, and that methylation on the maternal allele precludes this activity. Conformational analyses in an *in vitro* muscle differentiation model support this hypothesis, since they show that the MP is in close proximity to the repressed *Cdkn1c* promoter and this interaction is released upon differentiation (48).

**Figure 9.**
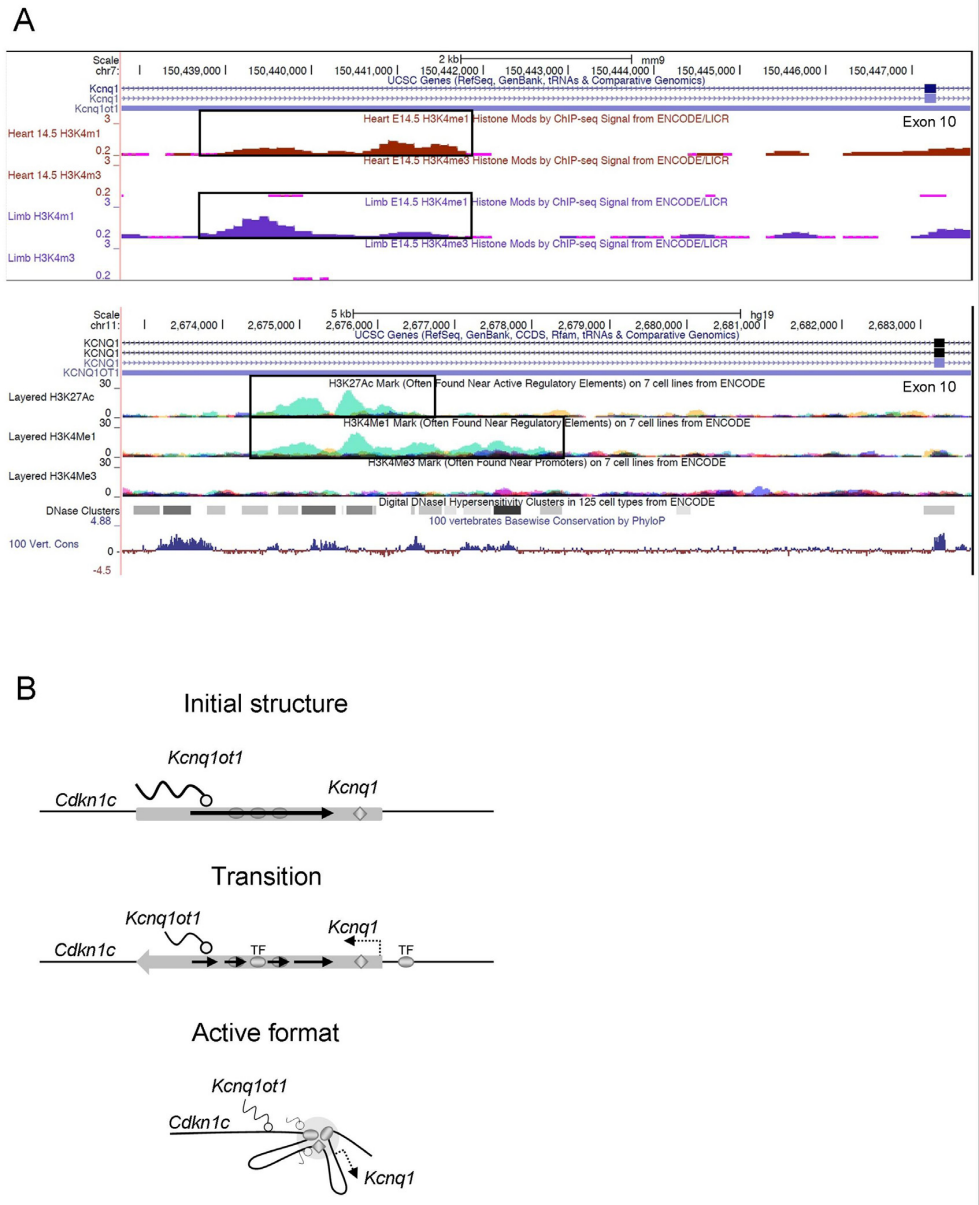
The *Kcnq1ot1* region encompasses multiple enhancers and may adopt distinct conformations to accommodate specific enhancer-gene interactions in different tissues. (**A**) Top, H3K4Me1 and H3K4Me3 profiles of the *Tbx5* binding site in mouse tissues as shown in the UCSC browser. Bottom, identification of a candidate enhancer in intron 10 of the human *Kcnq1* gene, highly homologous to the novel enhancer reported here. The image shows a partial region of the *Kcnq1* and *Kcnq1ot1* genes, with the H3K27Ac, H3K4Me1 and H3K4Me3 profiles of the candidate *Tbx5* enhancer. H3K4Me1 and H3K27Ac with low H3K4Me3 levels are frequently found at enhancer elements. (**B**) Model of heart-specific conformation as suggested by the 3C data. Ovals represent enhancers, diamond is the regulatory element active in early embryogenesis, wavy lines indicate RNAs. Width of lines represent levels of expression. Initially, *Kcnq1ot1* is highly expressed and tissue-specific enhancers are inactive. Between 11.5 and 14.5 dpc, the *Kcnq1ot1* and *Kcnq1* genes become biallelic, with *Kcnq1* levels increasing as transcription factors are expressed and bound to their cognate sites. *Kcnq1ot1* levels start to decrease and become discontinuous as, finally, the domain is compressed and restructured when enhancers are brought into proximity with the *Kcnq1* promoter.

The human *Kcnq1* domain is conserved in gene order, expression patterns and imprinting control with the mouse. Based on sequence conservation and histone modifications associated with enhancers (ENCODE), we identified a region in *Kcnq1* intron 10 overlapping with the *Kcnq1ot1* ncRNA that, upon analysis with rVISTA (49), was predicted to bind *Tbx5, Nkx2–5* and *MyoD*, among other cardiac transcription factors (Figure 9A), strongly pointing to heart-specific activity. Since *Kcnq1* is associated with long QT syndrome, it will be interesting to determine whether there are mutations in the non-coding portions of the gene that could be responsible for the cardiac phenotypes that have not been associated with mutations in the protein-coding regions.

There are a multitude of sequences within the *Kcnq1ot1* region exhibiting features of regulatory elements that need to be tested. We propose that the *Kcnq1ot1* region downstream of the *KvDMR* is a repository of enhancers and that the discontinuous levels of the ncRNA are due to activation of these enhancers by transcription factors. This leads to reorganization of the chromatin fiber, favoring induction of genes necessary for lineage determination at later stages. In the heart, for example, enhancers involved in cardiogenesis are engaged by the *Kcnq1* promoter, and this coincides with a maximum decrease in the abundance of *Kcnq1ot1* at 12.5 dpc, an up-regulation and loss of imprinting of *Kcnq1* and the compression of the whole domain (50,51) (Figure 9B). We also envision that some enhancers may not be functional *in vivo*, and that this region may be an evolutionary nursery for regulatory elements, as suggested by others (52). In a region as complex as the *Kcnq1* domain, with many genes active at different stages and in different tissues, insulators are likely to be important to ensure that genes are activated by the right enhancer. Ultimately we need to identify all the regulatory sequences, but also their relative locations, their tissue specificity and their three-dimensional architecture to fully understand their developmental role *in vivo*. The *Kcnq1* region is an excellent model to understand mechanistically how enhancers, insulators, ncRNAs and the target genes are deployed to generate the appropriate expression outputs in the endogenous context.

## SUPPLEMENTARY DATA

Supplementary Data are available at NAR Online.

SUPPLEMENTARY DATA
