# Supplementary material for: Enhancers compete with a long non-coding RNA for regulation of the Kcnq1 domain
Source: Nucleic Acids Res. 2014 Dec 24;43(2):745–59. doi: 10.1093/nar/gku1324 (PMC4333379; doi:10.1093/nar/gku1324)
Supplement: SUPPLEMENTARY DATA [file supp_43_2_745__index.html]

Enhancers compete with a long non-coding RNA for regulation of the Kcnq1 domain — SUPPLEMENTARY DATA 

# Enhancers compete with a long non-coding RNA for regulation of the *Kcnq1* domain

## SUPPLEMENTARY DATA

**Files in this Data Supplement:**

- SUPPLEMENTARY DATA
