# Supplementary material for: Enhancers compete with a long non-coding RNA for regulation of the Kcnq1 domain
Source: Nucleic Acids Res. 2014 Dec 24;43(2):745–59. doi: 10.1093/nar/gku1324 (PMC4333379; doi:10.1093/nar/gku1324)

**Supplementary Tables and Figures.**

**Supplementary Table 1.**

**Supplementary Figure 1**.

Summary of regions deleted in previous studies of the KvDMR, indicating status of *Cdkn1c* expression after paternal transmission of the deletions spanned by the arrows. None of those deletions retained the two *CTCF* binding sites purported to have insulator activity.


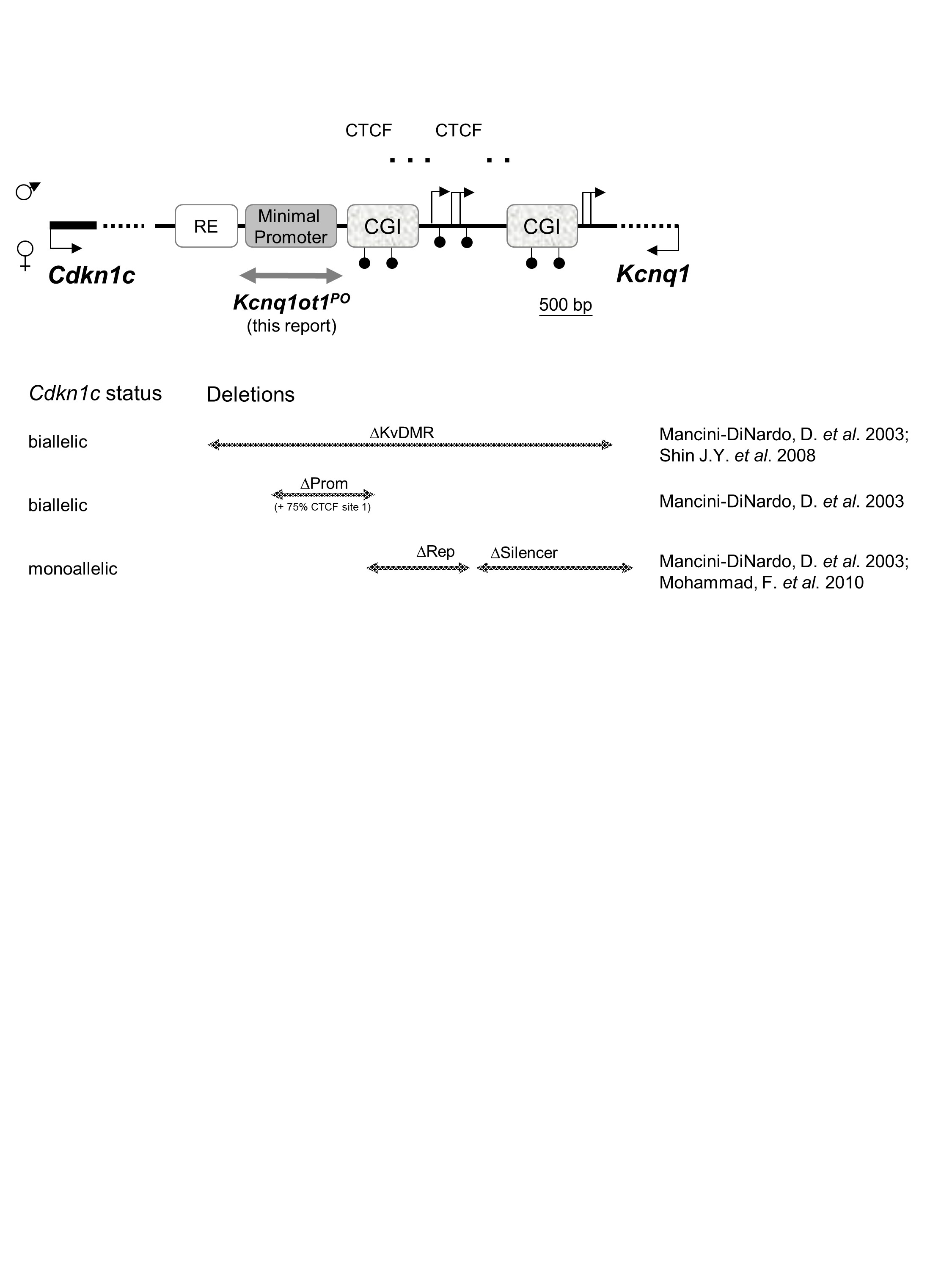


**Supplementary Figure 2**. Generation of the *Kcnq1ot1^PO^* allele.

A. Targeting strategy, illustrating from top to bottom the targeting vector (pTV-*Kcnq1ot1-MP*), the wild-type locus, and the correctly targeted alleles before (*Kcnq1ot1*^flox-MP-neoR^) and after excision of the *neo*R selection marker (*Kcnq1ot1*^PO^). HindIII sites used for screening are shown; the sizes of the fragments detected by Southern blot assays are indicated below the endogenous locus. Probes (A and B) are the bold horizontal lines. The endogenous sequences depicted are the *Kcnq1ot1* minimal promoter (MP) and the two CG islands (CGI); the vector includes loxP sites (white triangles) flanking the minimal promoter, PGK-*neo*R marker flanked by FRT sites (black triangles) and the 5’ and 3’ homology arms.

B. Southern blots to confirm correctly targeted alleles resulting from hybridization with labeled probes A and B after *Hind*III digestion of ES DNA. The fragments detected with probe A are the wild-type allele, 12.5 kb, and the recombinant allele, 7.8 kb. Probe B detects a 6.4 kb recombinant allele (arrows denote marker positions).

C. Long-range PCR with primers 1 and 2 (locations shown in panel A) confirming deletion of the *neo*R marker. Arrow indicates the 4.7 kb product.

D. Genotyping PCR for deletion of minimal promoter (MP). Primers 3 and 4 (indicated in panel A) yield a 700 bp product from the wild-type allele and a 292 bp product from the mutant allele.


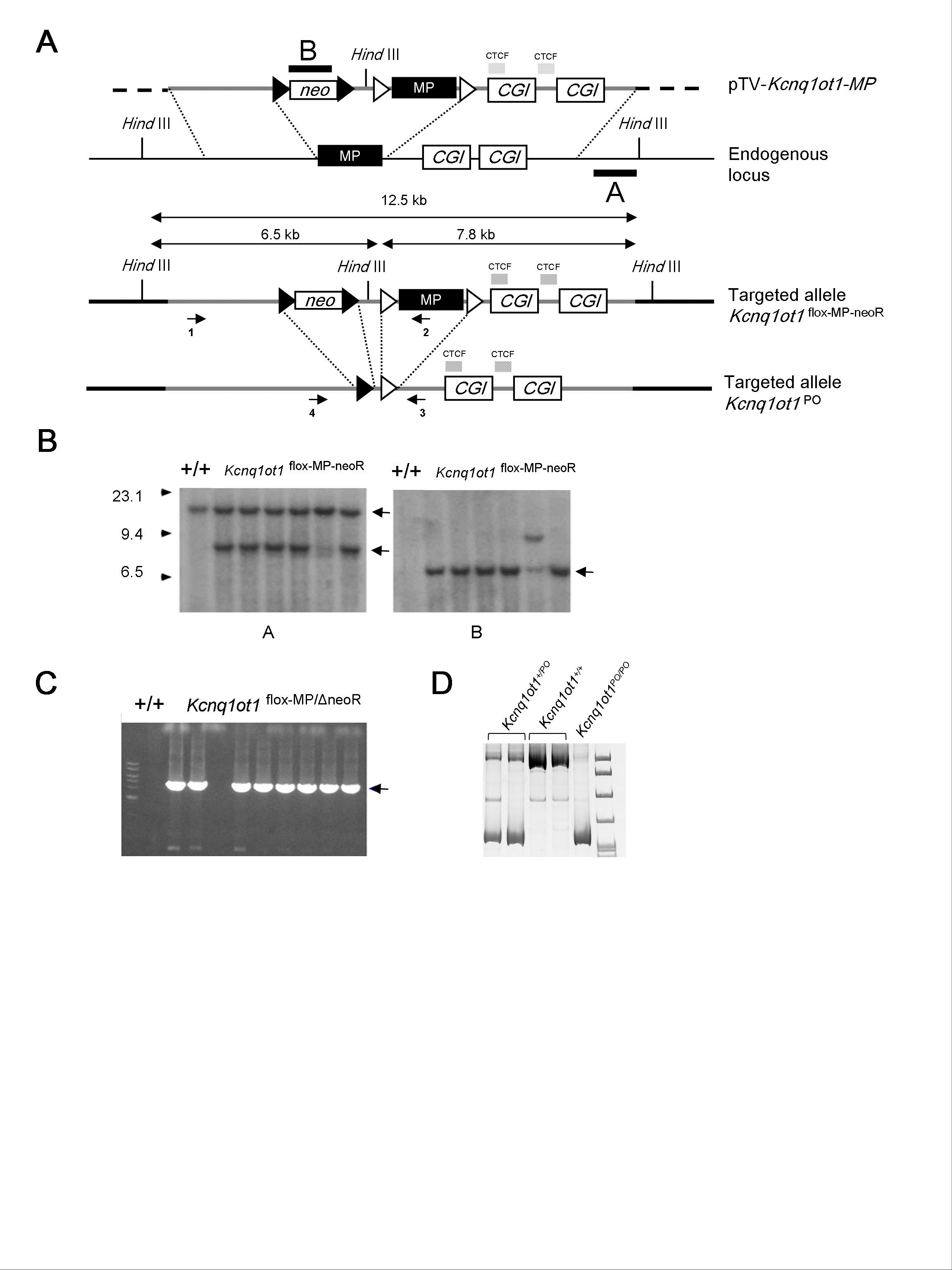


**Supplementary Figure 3.** Effects of the maternal inheritance of the *Kcnq1ot1^PO^* allele on imprinted expression of *Cdkn1c*. Combined analysis of *Cdkn1c* expression and imprinting in wild-type (WT) and *Kcnq1ot1^PO/+^* (MUT) mice (maternally inherited mutation) as assayed by qPCR and quantitative pyrosequencing, respectively, in neonatal hearts, brains and livers. Error bars indicate s.e.m.


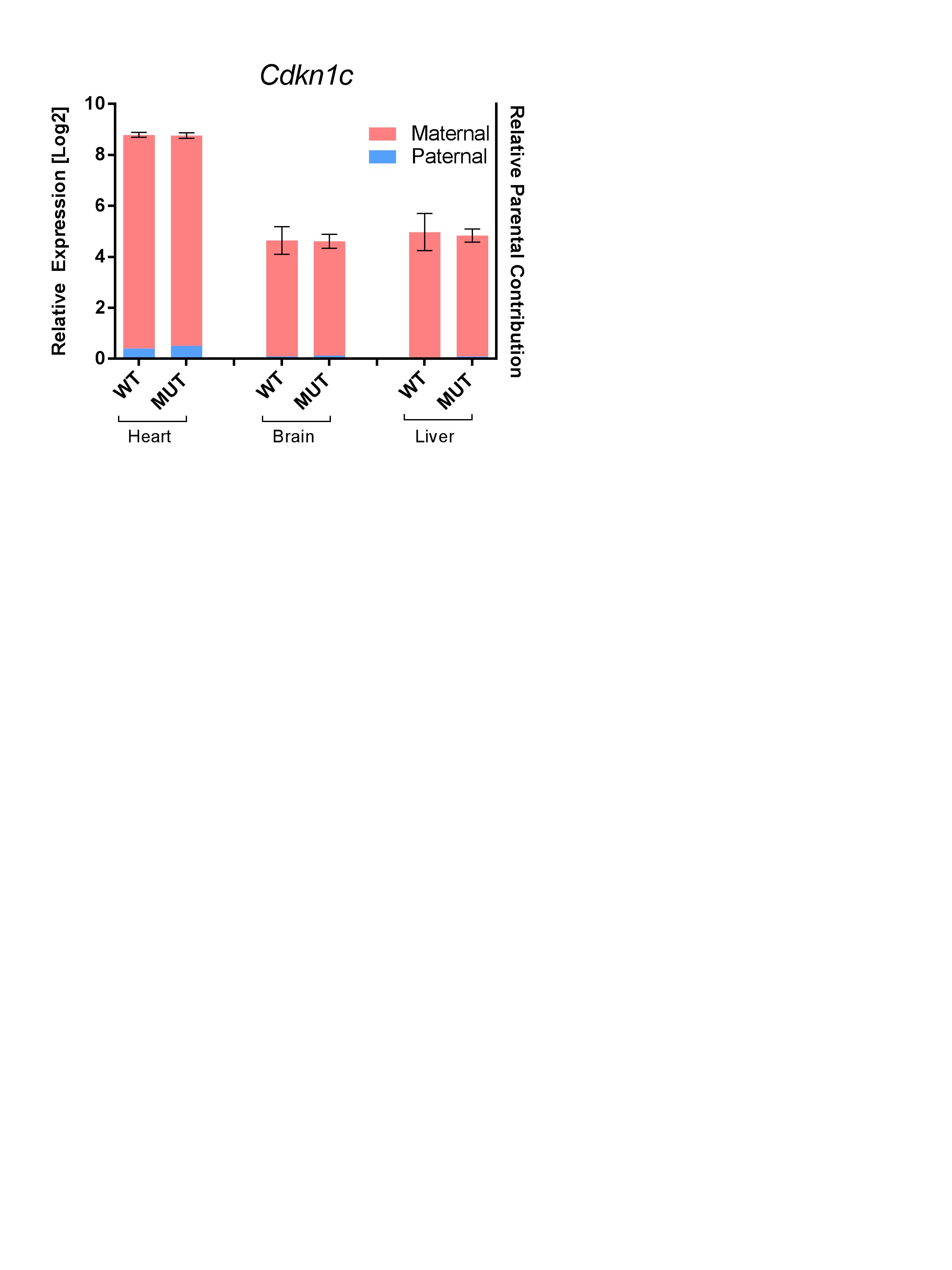


**Supplementary Figure 4.** Western blot with antibody to *Kcnq1* and β-actin for neonatal hearts from *Kcnq1ot1^+/+^* (WT) and *Kcnq1ot1^+/PO^* (MUT) mice, showing equal expression.


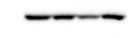

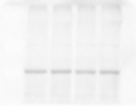


β-actin

Kcnq1

Neonatal Heart

WT

MUT

**Supplementary Figure 5.** Analysis of cardiac and brain *Kcnq1ot1* ncRNA imprinting in wild-type and *Kcnq1ot1^+/PO^* mice.

A. Schematic of *Kcnq1ot1* region (not to scale). Dotted arrow represents maternal transcription of *Kcnq1ot1* in the heart.

B. Combined analysis of *Kcnq1ot1* expression and imprinting neonatal heart (Nn Heart) in *Kcnq1ot1^+/+^* (top) and *Kcnq1ot1^+/PO^* mice (bottom) by qPCR and quantitative pyrosequencing. Primer sets were located 2, 33, 44, 60 and 90 kb from the minimal promoter (MP) to scan the entire coding region. Error bars indicate s.e.m. Asterisks above bars indicate a significant difference in RNA levels relative to those at 2 kb (for *Kcnq1ot1^+/+^*, 44 kb, p<0.0001; 90 kb, p=0.0015; for *Kcnq1ot1^+/PO^*, 44kb, p=0.0044 and 60kb, p<0.0001). Asterisks within the columns denote significant differences in parental contribution compared to 2kb (for *Kcnq1ot1^+/+^*, 44 kb, p<0.0001; for *Kcnq1ot1^+/PO^*, 33 kb, p=0.0018, 44, 60 and 90 kb, p<0.0001). The dotted box indicates the region in which expression is consistently diminished, coinciding with a candidate enhancer region (see Figure 7).

C. Combined analysis *Kcnq1ot1* expression and imprinting of brain (Nn Brain) in wild-type (top) and *Kcnq1ot1^+/PO^* mice (bottom) (assays as in B). Error bars indicate s.e.m. Asterisks above columns indicate a significant difference in RNA levels relative to levels at 2 kb downstream of the MP (for *Kcnq1ot1^+/+^*, 33 kb, p=0.0146; for *Kcnq1ot1^+/PO^*, 33 kb, p=0.0015; 60 kb, p=0.0248).


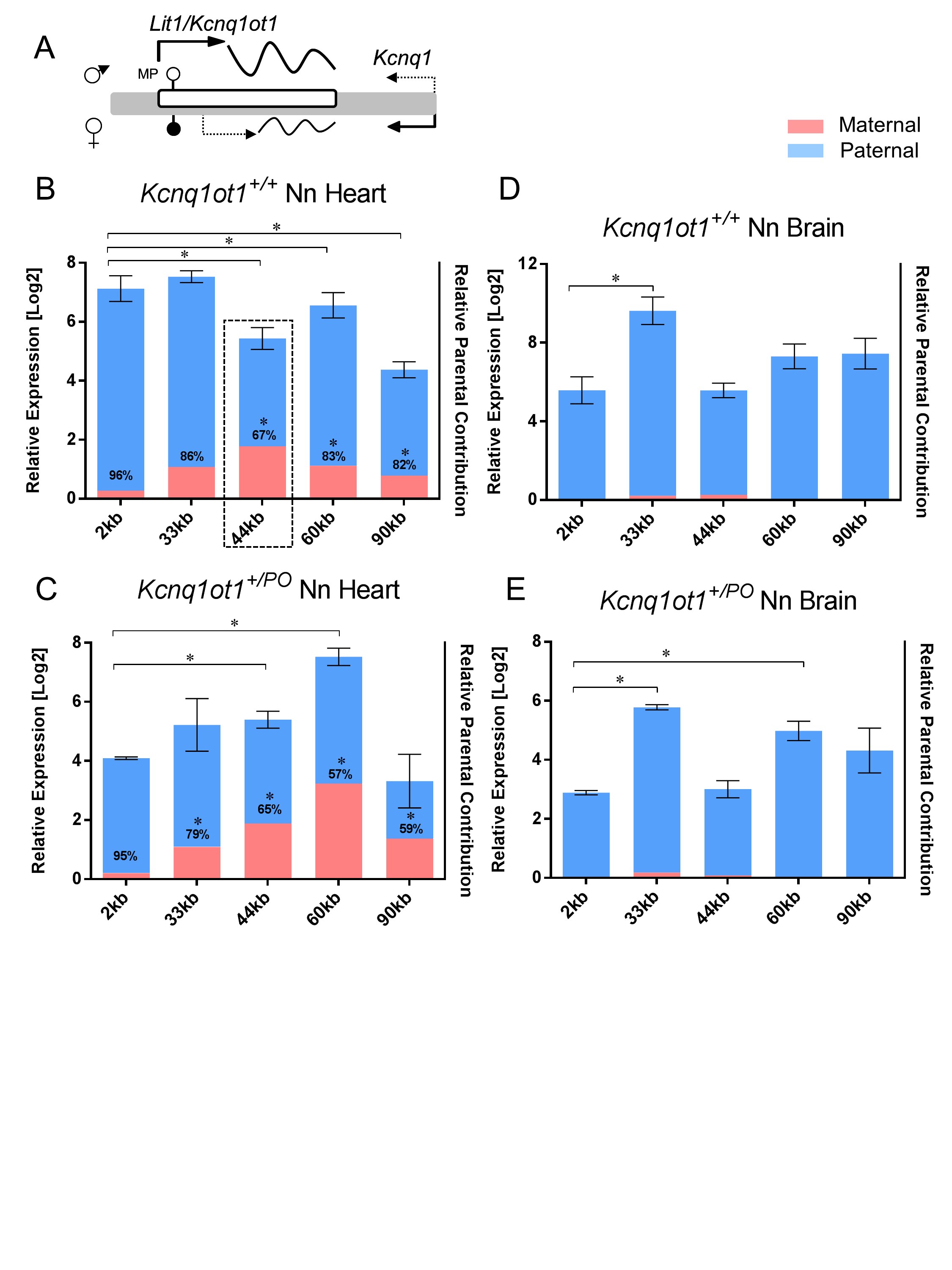


**Supplementary Figure 6.** Schematic showing the structure of the *Kcnq1ot1^+/PO^* allele. The regulatory element was characterized as an enhancer in vitro by Fitzpatrick et al.; the silencing domain (gray bar) was described by Mohammad et al. Primers used for 5’ RACE experiments are designated A and B, and nested primers, An and Bn. Biallelic heart-specific transcriptional start sites (TSS) are depicted as bent arrows. Stars indicate the relative location of single nucleotide polymorphisms (SNP) used to discriminate between parental transcripts. The first 25 bases of the novel transcript is indicated below.


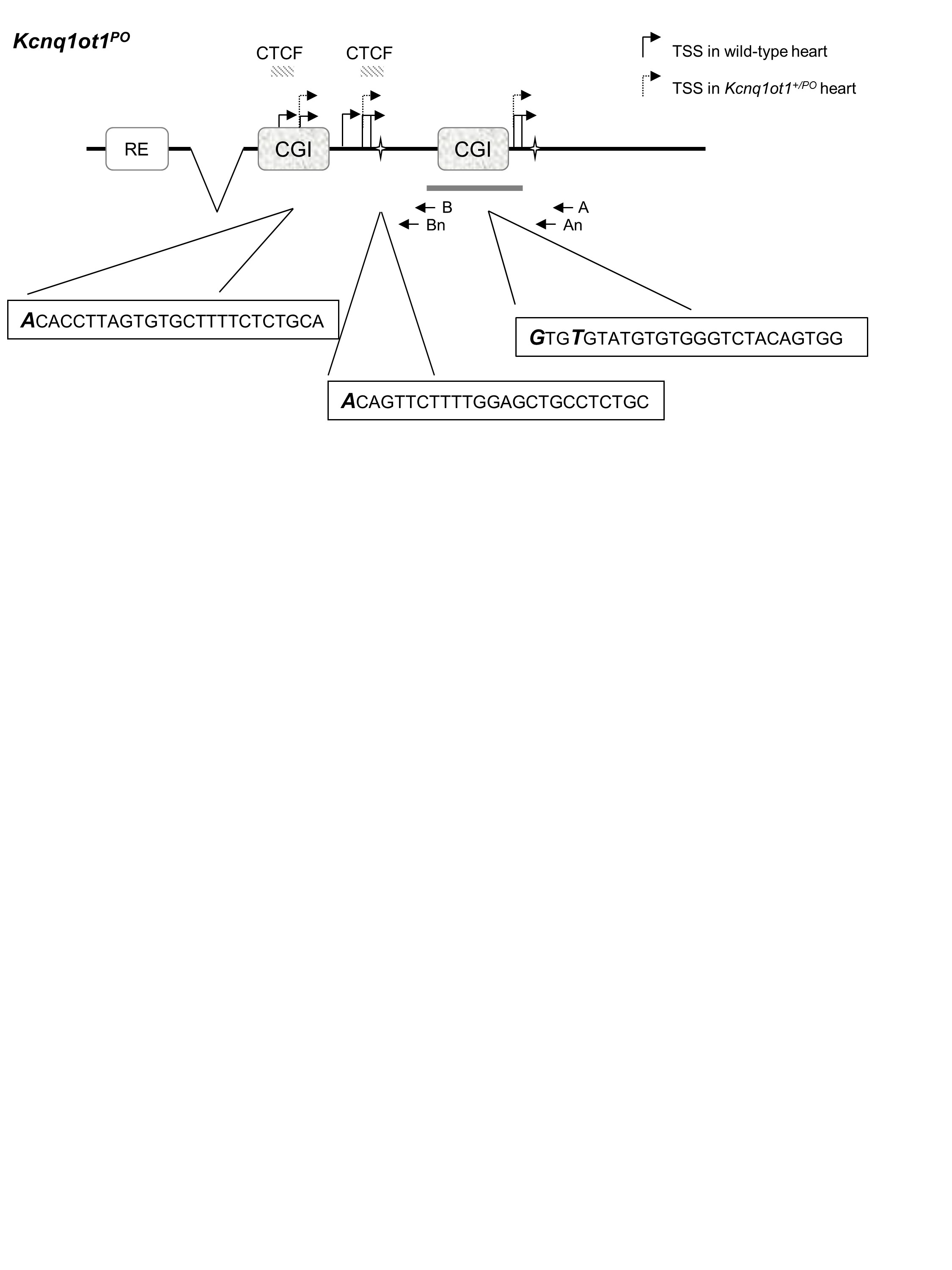


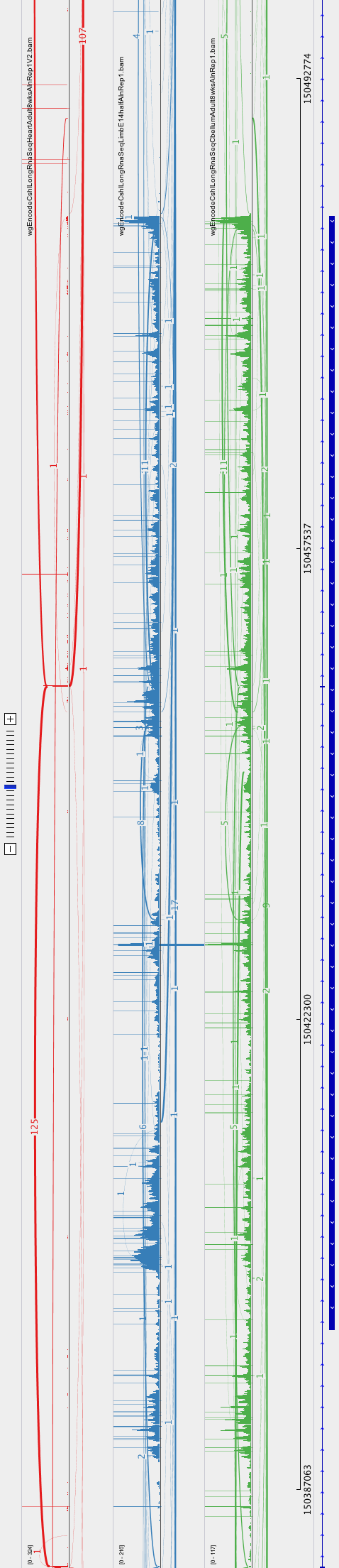


**Supplementary Figure 7.** Sashimi plot for two novel spicing events of the Kcnq1ot ncRNA. The graph plots the long RNA-sequencing mouse data downloaded from ENCODE and depicted in Figure 7A. The Sashimi plot allows quantitative visualization of mRNA sequencing reads aligned to gene annotations for exploratory analysis of alternatively spliced regions. Splice junctions are inferred from sequencing reads aligning to the 3’ and 5’ regions of different exons. Three tracks for three tissues are shown: adult heart (in red), 14.5 dpc limb (in blue) and adult brain (in green). Arcs indicate possible splice junctions and connect pairs of exons; arc width is proportional to the number of reads aligning to the junction, a measure of abundance of each isoform. Numbers indicated within the arc path are the actual number of identical reads aligning to two exons and spanning the intron.

**Supplementary Figure 8.** Functional validation of enhancer candidate. Top, lacZ-positive 11.5 transgenic embryos for *Tbx5* sequence. Bottom, negative control embryo obtained using empty vector, stained for lac-Z.


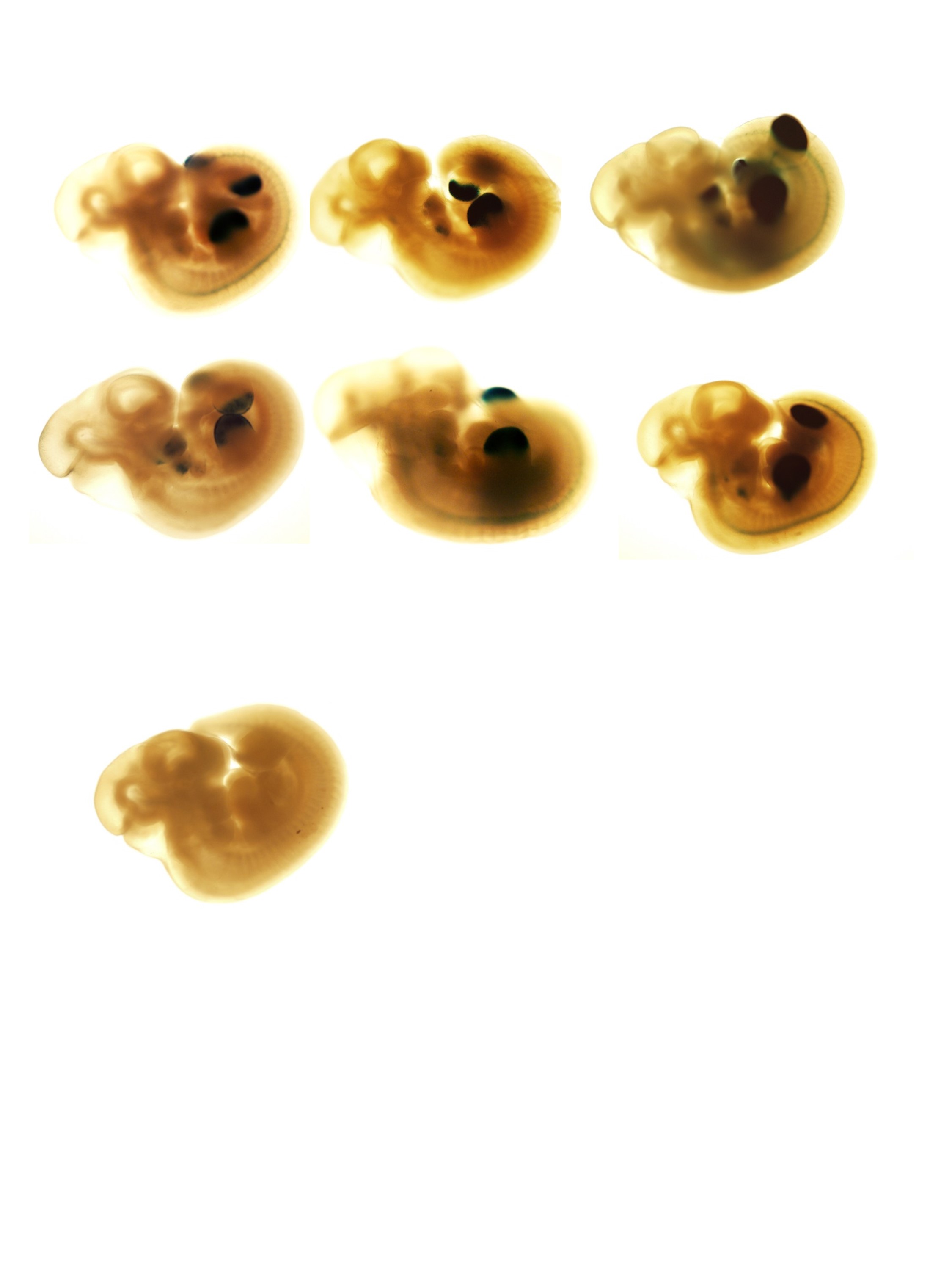

Supplement: SUPPLEMENTARY DATA [file supp_gku1324_nar-02729-a-2014-File011.doc]
